# Differences in the Structural Chemical Composition of the Primary Xylem of Cactaceae: A Topochemical Perspective

**DOI:** 10.3389/fpls.2019.01497

**Published:** 2019-11-28

**Authors:** Agustín Maceda, Marcos Soto-Hernández, Cecilia B. Peña-Valdivia, Carlos Trejo, Teresa Terrazas

**Affiliations:** ^1^Programa de Botánica, Colegio de Postgraduados en Ciencias Agrícolas, Texcoco, Mexico; ^2^Instituto de Biología, Universidad Nacional Autónoma de México, Mexico City, Mexico

**Keywords:** Fourier transform infrared, lignin, guaiacyl, topochemistry, primary xylem, vessel elements

## Abstract

The xylem of Cactaceae is a complex system with different types of cells whose main function is to conduct and store water, mostly during the development of primary xylem, which has vessel elements and wide-band tracheids. The anatomy of primary xylem of Cactaceae has been widely studied, but little is known about its chemical composition. The aim of this study was to determine the structural chemical composition of the primary xylem of Cactaceae and to compare it with the anatomy in the group. Seeds from eight cacti species were used, representing the Pereskioideae, Opuntioideae, and Cactoideae subfamilies. Seeds were germinated and grown for 8 months. Subsequently, only the stem of the seedling was selected, dried, milled, and processed following the TAPPI T-222 om-02 norm; lignin was quantified using the Klason method and cellulose with the Kurshner–Höffer method. Using Fourier transform infrared spectroscopy, the percentage of syringyl and guaiacyl in lignin was calculated. Seedlings of each species were fixed, sectioned, and stained for their anatomical description and fluorescence microscopy analysis for the topochemistry of the primary xylem. The results showed that there were significant differences between species (*p* < 0.05), except in the hemicelluloses. Through a principal component analysis, it was found that the amount of extractive-free stem and hot water-soluble extractives were the variables that separated the species, followed by cellulose and hemicelluloses since the seedlings developed mainly parenchyma cells and the conductive tissue showed vessel elements and wide-band tracheids, both with annular and helical thickenings in secondary walls. The type of lignin with the highest percentage was guaiacyl-type, which is accumulated mainly in the vessels, providing rigidity. Whereas in the wide-band tracheids from metaxylem, syringyl lignin accumulated in the secondary walls S2 and S3, which permits an efficient flow of water and gives the plant the ability to endure difficult conditions during seedling development. Only one species can be considered to have paedomorphosis since the conductive elements had a similar chemistry in primary and secondary xylem.

## Introduction

Xylem is a complex plant tissue formed by cells that carry out different functions (Baucher et al., 2007; Růžička et al., 2015) such as water conduction, support, and storage (Pratt et al., 2007; Brodersen and McElrone, 2013; Lucas et al., 2013). Secondary xylem is the most studied structure mainly in tree species (Danzer et al., 2001; Lupi et al., 2010), industrial pulping processes (Sahin and Arslan, 2008; Reddy et al., 2014), or timber-yielding production (Pawlicka and Waliszewska, 2011).

Seedlings of flowering plants have primary xylem, which is divided into protoxylem (initial stage) and metaxylem (stage previous to secondary growth), where vessel elements (VEs) are predominant (Bierhorst and Zamora, 1965; Carlquist and Schneider, 2010); nevertheless, in some angiosperm families, there are also tracheids (Feild et al., 2000) and wide-band tracheids (WBTs). The latter are imperforated elements with annular or helical thickenings of the secondary wall (Mauseth, 2004; Arruda and Melo de Pinna, 2010). The chemical composition of primary xylem has been described mainly in economically important herbaceous plants that do not have any secondary growth (Ekebafe et al., 2011; Cao et al., 2014), such as bamboo (Vena et al., 2010; Chang et al., 2013) and some forage species (Jung and Vogel, 1986; Cherney et al., 1988; Chaves et al., 2002; Tutt and Olt, 2011).

In Cactaceae family, the anatomical characteristics of the four subfamilies have been vastly studied (Mauseth and Plemons, 1995; Mauseth, 2004; Vázquez-Sánchez et al., 2017) because their stem morphological diversity is wide (tree, shrub, columnar, or globose; Vázquez-Sánchez et al., 2017), their size is diverse (small depressed globose to large trees; Vázquez-Sánchez and Terrazas, 2011), and their types of cellular matrices in the wood differ (monomorphic, dimorphic, and polymorphic; Mauseth and Plemons, 1998; Arnold and Mauseth, 1999) and are related to growth form (Gibson, 1978; Mauseth, 2006).

The chemical composition of secondary metabolites, analyzed by phytochemical profiling, has allowed the identification of the principal compounds in the stem (Loza-Cornejo et al., 2017), the structural chemical composition of stem conductive tissues (Reyes-Rivera et al., 2015; Maceda et al., 2018; Reyes-Rivera et al., 2018), and from other structures such as glochids and spines in several cacti species (Pritchard and Hall, 1976; Kohl et al., 2014).

The structural chemical composition of the secondary xylem of Cactaceae has been studied in fibrous, non-fibrous, and dimorphic species. The lignin concentration is the main component to distinguish fibrous species from non-fibrous ones (Maceda et al., 2018); also, the composition of lignin monomers is heterogeneous in non-fibrous and dimorphic species, but the occurrence of syringyl and guaiacyl is homogeneous in fibrous species (Reyes-Rivera et al., 2015; Reyes-Rivera et al., 2018). In addition, cellulose and lignin percentages differ with other families and between Cactaceae genera (Maceda et al., 2018).

In the case of primary xylem of Cactaceae, little is known about the chemical composition and proportion of the structural compounds of its cell elements. Several authors have suggested that Cactaceae wood is paedomorphic (Altesor and Ezcurra, 2003; Mauseth, 2004; Dulin and Kirchoff, 2010), related to the occurrence of WBTs in adult age or having WBTs only in the juvenile stage (Loza-Cornejo et al., 2003; Mauseth, 2004; Loza-Cornejo and Terrazas, 2011), changing to fibers in the adult one (Gibson, 1978; Mauseth and Plemons, 1998). Because of this, knowledge on the structural chemical composition of the primary xylem will allow us to establish similarity between primary and secondary xylem and to support the hypothesis of paedomorphism in the xylem tissue of this family. Therefore, in the present study, we determine the chemical composition of the primary xylem in seedlings of eight Cactaceae species, we obtain infrared spectra of lignin to calculate the syringyl/guaiacyl proportion, describe the vascular tissue with fluorescence microscopy, and compare the chemical composition of primary and secondary vascular tissues of the same species.

## Materials and Methods

### Plant Material

Ripe fruits from eight Cactaceae species were collected in several regions of Mexico, which represent the different types of wood and growth forms (**Table 1**). The fruits were dissected to obtain seeds. *Echinocactus platyacanthus*, *Ferocactus pilosus*, *Leuenbergeria lychnidiflora*, *Lophocereus marginatus*, *Mammillaria carnea*, and *Myrtillocactus geometrizans* seeds were disinfected by immersion in 10% sodium hypochlorite for 5 min, placed on filter paper saturated with distilled water in sterile Petri dishes (Loza-Cornejo et al., 2008; Guillén et al., 2011), and kept at 25°C under a light/darkness photoperiod of 12 h in a germination chamber (Rojas-Aréchiga and Mandujano, 2013; Salas-Cruz et al., 2014; Bevilaqua et al., 2015). *Cylindropuntia imbricata* and *Opuntia streptacantha* seeds were scarified with 98% sulfuric acid for 90 min, washed five times with distilled water, disinfected by immersion in 10% sodium hypochlorite for 5 min, and placed on filter paper saturated with distilled water in sterile Petri dishes (Sánchez-Soto et al., 2010; Monroy et al., 2017). Seeds of both Opuntioideae and *L. lychnidiflora* were also kept in a germination chamber at 30°C and under a light/darkness photoperiod of 12 h (Souza et al., 2016).

**Table 1:** Morphology and stem characteristics of eight Cactaceae species.

Species	Wood type (adult stage)	Stem	Plant size
*Echinocactus platyacanthus* Link & Otto	Dimorphic	Columnar	Medium
*Ferocactus pilosus* Briton & Rose	Dimorphic	Columnar	Medium
*Cylindropuntia imbricata* (Haw.) F.M. Knuth	Fibrous	Tree	Tall
*Leuenbergeria lychnidiflora* (DC.) Lodé	Fibrous	Tree	Tall
*Lophocereus marginatus* (DC.) S. Arias & Terrazas	Fibrous	Tree	Tall
*Mammillaria carnea* Zucc. ex Pfeiff.	Non-fibrous	Globose	Small
*Myrtillocactus geometrizans* (Mart. ex Pfeiff.) Console	Fibrous	Tree	Tall
*Opuntia streptacantha* Lem.	Fibrous	Tree	Tall

After 30 days of germination, the seedlings were transplanted to trays with a 1:1:1 mixture of perlite–tezontle–potting soil and kept in the conditions previously described for germination under a two-times-per-week water regime (Loza-Cornejo and Terrazas, 2011; Bárcenas-Argüello et al., 2013). From 2 to 8 months of age, we selected the seedlings [classified as such because they maintained cotyledons (**Figure S1**) and showed abundant primary vascular tissue] (Loza-Cornejo and Terrazas, 2011) for evaluations.

### Standardization of the Method

For each collected seedling, the spines were eliminated to avoid their lignin interfering with the vascular tissue analysis, then seedlings were desiccated at 50°C for 48 h and milled using a mortar until their particle size allowed them to go through a 0.4- to 0.6-mm mesh. The extractive-free lignocellulose was obtained from these samples (Wahab et al., 2013; Reyes-Rivera and Terrazas, 2017).

The amount of sample required to extract and quantify extractives, Klason lignin, cellulose, and hemicellulose was adapted from the methodology of the TAPPI T-222 om-02 norm. This method has been used in studies of wood (Dence, 1992; Wahab et al., 2013) and fibers (Han and Rowell, 1996), including adult plants of Cactaceae (Reyes-Rivera et al., 2015; Maceda et al., 2018). For each one of the eight species, 75 seedlings were dried and milled and the material was homogenized. We used 25 seedlings per replicate and three replicates per species to perform the extraction and quantification procedure.

#### Stem Extractives

0.2 g of each milled stem sample was placed in a filter paper cartridge, kept for 12 h at 60°C, and its weight was registered. The extractive-free stem samples were obtained by successive extractions in Soxhlet (Reyes-Rivera and Terrazas, 2017), with ethanol/benzene (1:2, *v*/*v*) and 96% ethanol; each of the extractions took 24 cycles, which were 5 h approximately. The cartridges were left to dry for 12 h to obtain constant weight (Maceda et al., 2018). The samples were removed from the cartridges and they were extracted in distilled water for an hour at 95°C, filtered through a medium-pore Büchner filter, and kept at 105°C for 12 h and their constant weight was recorded (Maceda et al., 2018). With the weights of the residues after each extraction, the percentage of stem extractives was calculated according to the following formula:

$$\%=[\left(A+B+C\right)/W_{0}]*100$$

where *A* is the weight (in grams) after the ethanol/benzene extraction, *B* the lost weight (in grams) after the ethanol extraction, *C* the lost weight (in grams) after the warm water extraction, and *W*_0_ is the weight of the initial sample (in grams).

#### Klason Lignin

It was quantified by the method of the TAPPI T-222 om-02 norm (Abreu et al., 2004; Latorraca et al., 2011; Fonseca-Prieto et al., 2014), with some modifications. Of 72% sulfuric acid (in water, *v*/*v*), 0.5 ml was added to 0.05 g of the extractive-free sample and kept at 18°C for 2 h and shaken every 30 min. This allowed the sample to completely soak in the solution. After this, 14 ml of distilled water was added and it was kept at boiling point and at a constant volume for 4 h. The residue, which includes lignin, was filtered through a Büchner crystal funnel with fine pore, dried at 105°C for 12 h, and the constant weight of the sample was recorded (Maceda et al., 2018). Lignin percentage was determined according to the following formula:

$$\%\;\mathrm{lignin}=\left(W_{\text{L}}/W_{\text{W}}\right)*100$$

where *W*_L_ is the weight (in grams) of the resultant lignin and *W*_W_ is the weight (in grams) of the extractive-free stem sample.

#### Cellulose and Hemicellulose

Kûrshner–Höffer’s method (Abreu et al., 2004) was modified to quantify cellulose. From extractive-free stem, 0.05 g was weighed and 1.25 ml of HNO_3_–ethanol (1:4, *v*/*v*) was added. A boiling-reflux system was mounted in a water bath for 1 h. The HNO_3_–ethanol reactive solution was eliminated by decantation and 1.25 ml of HNO_3_–ethanol was added again. The extraction was made three times and in the last decantation, 1.25 ml of aqueous 1% KOH solution was added, the reflux was kept for 30 min, and it was finally filtered out through a Büchner filter. The residue was left to dry for 12 h at 50°C and the cellulose constant weight was recorded (Abreu et al., 2004; Maceda et al., 2018). Cellulose percentage was obtained according to the following formula:

$$\%\;\mathrm{cellulose}=\left(\frac{W_{\text{C}}}{W_{\text{W}}}\right)*100$$

where *W*_C_ is the cellulose weight (in grams) and *W*_W_ is the extractive-free stem sample weight (in grams).

Hemicellulose percentage was determined with the gravimetric method proposed by Li et al. (2017), with few modifications. From extractive-free stem, 0.5 g was weighed and extracted with a reflux system with 10 ml of hot water for 3 h (solid-to-liquid ratio, 1:20 ml). The reaction system was cooled to room temperature and filtrated. The filtrated was concentrated to 1.25 ml and was poured into 3.75 ml of 95% ethanol with stirring. The mixture was placed for 1 h and hemicellulose precipitate was obtained by centrifugation (4,500 × *g* for 4 min) and freeze drying. The constant weight was recorded (*H*_0_). The water-insoluble solid residue was dried at 60°C for 16 h in an oven. After that, the solid residue was stepwise extracted with different concentrations of KOH (0.6%, 1.0%, 1.5%, 2.0%, and 2.5%) at 75°C for 3 h under a ratio of 1:20 (in grams per milliliter). In a final step (2.5% of KOH), the solution of 2.5% of KOH was mixed with ethanol (99.7%) in a 2:3 ratio. The five mixtures were filtered and then the filtrates were acidified to pH 5.5 with glacial acetic acid and concentrated to 1.25 ml. The mixtures were poured in 3.75 ml of 95% ethanol with vigorous stirring; then, the mixture was placed for 1 h to precipitate the hemicellulose and was finally obtained by centrifugation (4,500 × *g* for 4 min) and freeze drying. The constant weight was recorded in each step (*H*_0.6_, *H*_1.0_, *H*_1.5_, *H*_2.0_, and *H*_2.5_). Total hemicellulose was obtained by the following formula:

$$\%\;\mathrm{hemicellulose}=\left(\frac{W_{\text{H}}}{W_{\text{W}}}\right)*100$$

### Syringyl/Guaiacyl Ratio Lignin

The estimation of lignin composition has been made through different methods: pyrolysis–gas chromatography–mass spectrometry (Py-GC/MS) (Klap et al., 2000), nuclear magnetic resonance (NMR) (Jin et al., 2007; Weng et al., 2010; Marques et al., 2016; Reyes-Rivera et al., 2018), Fourier transform infrared (FTIR) (Li et al., 2015; Reyes-Rivera et al., 2015; Reyes-Rivera et al., 2018), and high-performance liquid chromatography (HPLC) (Jaramillo-Carmona et al., 2008; Kline et al., 2010; Reyes-Rivera et al., 2015). For this reason, in this paper, Klason lignin is used directly to perform infrared (FTIR) spectrum readings, due to Reyes-Rivera et al. (2018) finding consistent values when comparing these with other methods such as Py-GC/MS and NMR. Therefore, after the Klason lignin was obtained from the seedlings, the samples were kept in a desiccator and FTIR-ATR spectra were obtained using an Agilent Cary 630 FTIR-ATR equipment. Ten spectra were obtained for each sample. Their average was calculated and then the baseline and ATR corrections were performed to diminish spectrum noise (Jääskeläinen et al., 2003; Zhou et al., 2011b; Reyes-Rivera et al., 2018). After that, the 1,269- to 1,272-cm^−1^ peaks were used to measure guaiacyl values and the 1,328- to 1,330-cm^−1^ peaks to measure syringyl values (Luna et al., 2015; Safou-Tchiama et al., 2017). The measurement was made following Pandey’s method (Pandey’s, 2005) in which, after baseline correction, the values of the peaks are measured by creating a line that connects the lower and the higher values of the peak; a vertical line is drawn from the base of the *x*-axis to the top of the peak. The portion of the line between the top of the peak and the baseline is the correct value for each peak. Once the values of every syringyl and guaiacyl peak were obtained, the percentages of each monomer were divided to obtain the syringyl/guaiacyl (S/G) ratio.

### Statistical Analysis

Coefficient of variation (CV) was used as a measure to quantify the method’s repeatability (Chaves et al., 2002) because values smaller than the TAPPI norm were used. The CV was obtained with the following formula:

$$\mathrm{CV}\left(\%\right)=\left(\frac{\mathrm{SD}}{\mu }\right)*100$$

where SD is the sample standard deviation and *µ* is the repetition average of each sample.

Once the mean, standard deviation, and CV were obtained, normality was determined by the Kolmogorov–Smirnov test and the Shapiro–Wilk analysis, which showed that there was no normality in the data, even after using the arcsine square root. Therefore, the non-parametric Kruskal–Wallis test was applied, as well as the Dunn test as *post hoc* which compares multiple pairs between each species and determines if the differences in the variables among the eight species were significant (Dinno, 2015). The results were compared with those reported by Reyes-Rivera et al. (2015) and Maceda et al. (2018), and with studies that include primary xylem data (**Table S1**).

Then, two principal component analyses (PCA) were performed—the first one to define the variables that determined the differences between seedlings and the second one with the variables of the seedlings and the adult plants of each species using the values of Reyes-Rivera et al. (2015) and Maceda et al. (2018).

### Anatomical and Topochemical Description of Lignin

Two to four seedlings per species were used for the anatomical description to obtain transverse and longitudinal sections. The tissues were fixed in formaldehyde, acetic acid, and alcohol and kept in a vacuum chamber for 24 h. This procedure facilitates the infiltration of the fixer and avoids vascular tissue collapse (Huang and Yeung, 2015). The tissues were embedded in paraffin, sections cut 10–12 µm thick using a rotary microtome, dewaxed, and stained with safranin O/fast green (Loza-Cornejo and Terrazas, 1996).

Microscopy fluorescence combined with safranin O/fast green can be useful to identify differences in vascular tissue composition (Haseloff, 2003; De Micco and Aronne, 2007; Frank et al., 2007) because safranin O contributes to highlighting the fluorophores present in the lignin molecule (Angeles et al., 2004; Bond et al., 2008; Donaldson, 2013; Zeng et al., 2015). This combination has a greater sensitivity to identify variations in the composition of vascular tissue compared with Maüle and Wiesner techniques (Kutscha and McOrmond, 1972; Decou et al., 2017; Kiyoto et al., 2018). Lignin fluorescence has been characterized by a wide range of excitation and emission wavelengths (Donaldson et al., 2010; Donaldson, 2013; Zeng et al., 2015). It has been proposed to use different wavelengths to identify variations in the intensity and tonalities of fluorescence lignin (Angeles et al., 2004; Ji et al., 2013; Zeng et al., 2017; Donaldson and Williams, 2018). Therefore, the observations were made on a fluorescence microscope with the DAPI–FITC–TRITC band excitation, which has three types of waves: violet (385–400 nm), blue (475–490 nm), and green (545–565 nm), with emission filters of 430, 510, and 570 nm, respectively. For this, each sample was kept for 1 min under exposure to observe the autofluorescence of lignin (Donaldson and Williams, 2018) highlighted by safranin O (Dürrenberger et al., 2001; Angeles et al., 2004; Bond et al., 2008).

## Results

### Structural Chemical Composition

The modifications made to the TAPPI norm allowed the repeatability of extractives, cellulose, lignin, and hemicellulose percentages (**Tables 2** and **3**). The CV of the percentages of extractives was less than 5%, except for one *O. streptacantha* variable (water at 90°C) that had a CV above 5% (**Table 2**). Hemicelluloses showed CV values less than 5%. With respect to lignin, four species showed a CV slightly above 5%, but only one species showed this value for cellulose (**Table 3**).

**Table 2:** Average of the extractives in seedlings of eight species of Cactaceae.

Species	Ethanol/benzene (%)	CV	Ethanol 96% (%)	CV	Water 90°C (%)	CV
*C. imbricata*	7.8 ± 0.2	2.6	3.8 ± 0.2	4.2	7.6 ± 0.2	3.1
*E. platyacanthus*	8.5 ± 0.3	3.4	4.4 ± 0.1	3.2	10.4 ± 0.1	1.1
*F. pilosus*	9.7 ± 0.4	3.9	5.2 ± 0.2	4.3	9.7 ± 0.1	0.6
*L. lychnidiflora*	4.3 ± 0.1	2.4	2.5 ± 0.1	3.7	5 ± 0.1	1.9
*L. marginatus*	4.8 ± 0.2	4.4	4.4 ± 0.2	4.3	7.5 ± 0.4	4.8
*M. carnea*	8.2 ± 0.4	4.7	5.7 ± 0.2	3.5	9.8 ± 0.1	0.9
*M. geometrizans*	7 ± 0.1	1.7	4.7 ± 0.1	2.7	6.5 ± 0.3	4.1
*O. streptacantha*	7.5 ± 0.3	3.6	5.1 ± 0.2	3.7	9.1 ± 0.8	8.6

CV, coefficient of variation.

**Table 3:** Average of the lignocellulose percentages in seedlings of eight Cactaceae species.

Species	Extractive-free stem (%)	CV	Cellulose (%)	CV	Lignin (%)	CV	Hemicellulose (%)	CV
C. *imbricata*	80.7 ± 0.6	0.7	53 ± 2.2	4.1	10 ± 0.5	4.8	19.5 ± 0.2	1.3
*E. platyacanthus*	76.8 ± 0.5	0.7	52.8 ± 3.2	6.1	7.8 ± 0.2	2.7	19.2 ± 0.4	1.8
*F. pilosus*	75.4 ± 0.7	0.9	52.9 ± 0.7	1.4	7.7 ± 0.3	3.4	18.3 ± 0.4	2.4
*L. lychnidiflora*	88.2 ± 0.3	0.3	55.8 ± 0.9	1.6	11.5 ± 0.8	6.7	17.7 ± 0.5	2.6
*L. marginatus*	83.2 ± 0.7	0.9	56.2 ± 1.4	2.5	9.9 ± 0.6	6.3	18.2 ± 0.4	2.2
*M. carnea*	76.5 ± 0.7	0.9	54.5 ± 0.7	1.3	7.4 ± 0.6	7.5	19.5 ± 0.3	2
*M. geometrizans*	81.8 ± 0.5	0.6	54.6 ± 1.3	2.4	10.6 ± 0.4	3.9	18 ± 0.4	2.4
*O. streptacantha*	78.4 ± 1.2	1.6	51.3 ± 1.8	3.4	9.8 ± 0.7	6.7	19.1 ± 0.4	2.2

CV, coefficient of variation.

**Figure 1:**
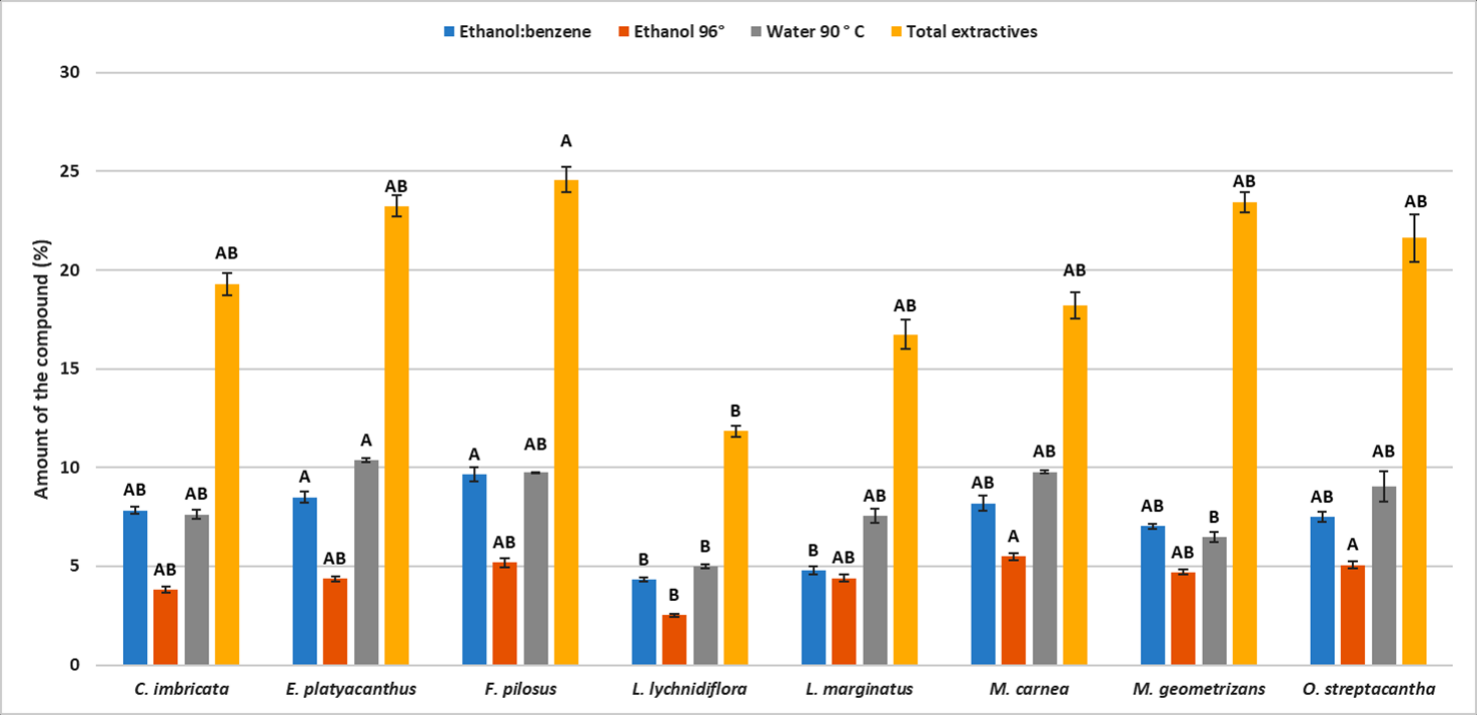
Mean of the percentages for each type of extractive in eight Cactaceae species. *Different letters* indicate significant differences between means based on pairwise multiple comparison Dunn’s test (*p* ≤ 0.05). *Bars* represent standard deviation.

**Figure 2:**
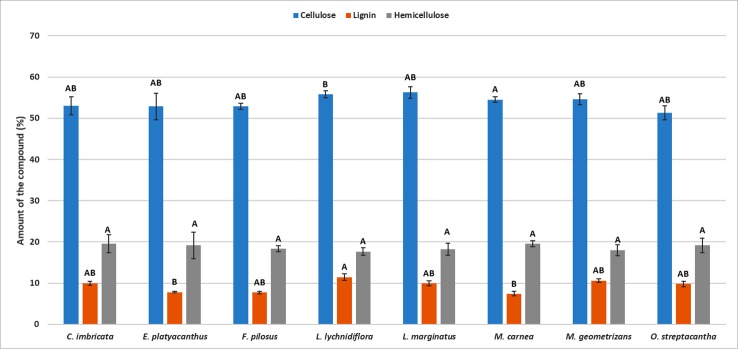
Mean of the percentages for each lignocellulosic compound in eight Cactaceae species. *Different letters* indicate significant differences between means based on pairwise multiple comparison Dunn’s test (*p* ≤ 0.05). *Bars* represent standard deviation.

The differences in the content of structural components were significant (*p* < 0.05) among the species—the only exception being hemicellulose (*p* = 0.09, **Table S2**). Furthermore, the *post hoc* Dunn’s test showed that the diff-erences among extractives of *L. lychnidiflora* (B) and the rest of the species were significant (**Figure 1**). Cellulose proportion varied significantly between *M. carnea* (A) and *L. lychnidiflora* (B) and the lignin proportion of *L. lychnidiflora* (A) with *E. platyacanthus* and *M. carnea* (B) (**Figure 2**).

### Principal Component Analysis

**Figure 3:**
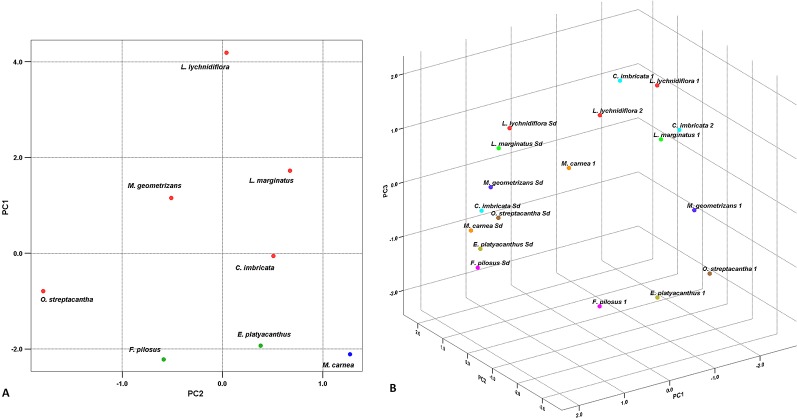
Principal component analysis. **(A)** Graphic of the eight Cactaceae seedlings based on the first two principal components. The *red dots* represent the fibrous species, the *green dots* the dimorphic species, and the *blue dot* the non-fibrous species. **(B)** Dispersion graphic in the eight species of seedlings and adults. *Colors* correspond to each distinct species. *Sd* seedling. *1* Adult plant from Reyes-Rivera et al. (2015). *2* Adult plant from Maceda et al. (2018).

The PCA for seedlings showed that the first two principal components explain 85% of the total variation between species (**Figure 3A**). In the first component (PC1), the extractive-free stem and extractives in water at 90°C showed the highest contribution (**Table 4**); this was the case for cellulose and hemicellulose in the second component (PC2). In the graphic representation, *L. lychnidiflora* was separated from the other species (**Figure 3A**).

**Table 4:** Eigenvectors, eigenvalues, and accumulated proportion of the explained variation by each variable.

Variables	Seedlings		Seedlings and adult plants		
	PC1	PC2	PC1	PC2	PC3
Ethanol/benzene	−0.41	−0.14	0.24	0.05	−**0.75**
Ethanol 96°C	−0.36	−0.13	0.31	−0.35	0.53
Water 90°C	−**0.42**	0.04	0.19	−**0.61**	−0.06
Extractive-free wood	**0.44**	0.07	−0.31	0.53	0.21
Cellulose	0.3	**0.61**	**0.46**	0.37	0.07
Lignin	0.4	−0.29	−**0.53**	−0.18	0.13
Hemicellulose	−0.27	**0.71**	0.46	0.23	0.29
Eigenvalues	5.08	0.89	3.01	2.26	1.38
Proportion variance (%)	72.5	12.8	43	32.3	19.7
Accumulated variance (%)	72.5	85.3	43	75.3	95

Bold numbers are the variables with the highest contribution within the PC.

In the PCA for seedlings and adults, lignin and cellulose had the strongest influence in total variation in the PC1 (**Table 4**), while extractives in water at 90°C (PC2) and ethanol–benzene extractives (PC3) also contributed with variability, but in a smaller proportion. The graphic representation of the three components showed the separation between seedlings and adult plants (**Figure 3B**).

### Chemical Composition of Lignin of the Primary Xylem

**Figure 4:**
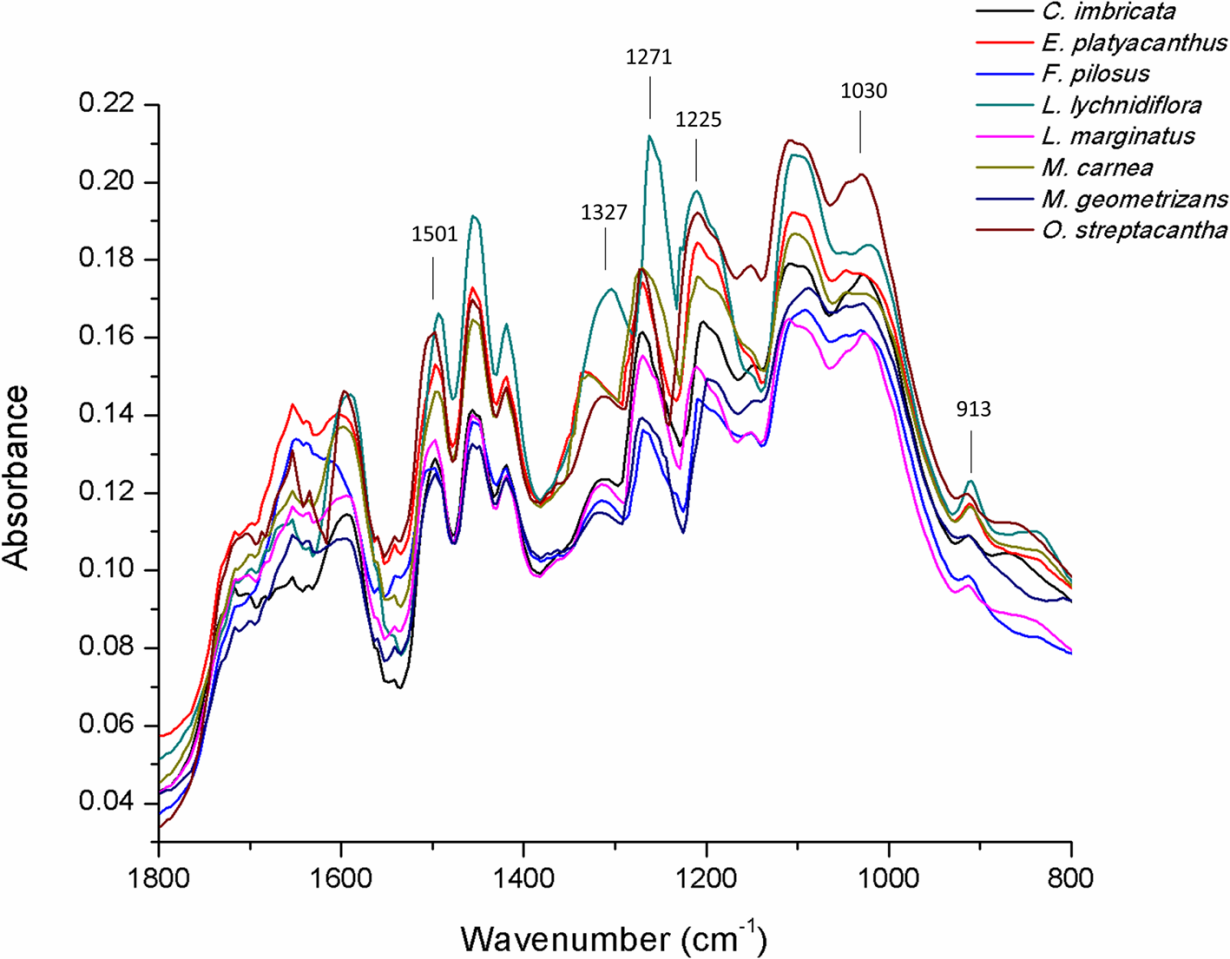
FTIR-ATR spectrum of lignin in the primary xylem of seedlings of eight Cactaceae species.

The results of FTIR-ATR spectrum (**Figure 4**) showed the fingerprint peaks for lignin (800–1,800 cm^−1^). The peak associated with 1,501 cm^−1^ was the C=C aromatic ring vibration of guaiacyl–syringyl. At 1,325 cm^−1^, the syringyl ring breathing with C–O stretching was detected, whereas the peak at 1,271 cm^−1^ detected the C–O and glucopyranosic cycle guaiacylic symmetric vibration. The peak at 1,225 cm^−1^ detected C–O and glucopyranosic cycle syringylic symmetric vibration, and the peak at 1,030 cm^−1^ detected the C–H in-plane deformation in guaiacyl and C–O deformation in primary alcohol. The last peak at 913 cm^−1^ was the =CH out-of-plane deformation in aromatic ring (guaiacylic–syringylic).

With peaks at 1,325 and 1,271 cm^−1^, the primary xylem ratio S/G from the stem was calculated (**Table 5**). The syringyl proportion was different among the species. *M. geometrizans* showed the lowest percentage (20.67%) and *L. lychnidiflora* the highest one (41.87%) of the whole group. On the contrary, guaiacyl proportion had the lowest value in *L. lychnidiflora* (58.13%) and the highest one in *M. geometrizans* (79.33%).

**Table 5:** Syringyl and guaiacyl values for primary xylem compared to secondary xylem of eight Cactaceae species.

Species	S (%)	G (%)	S/G ratio	S (%)	G (%)	S/G ratio
	Seedlings (Primary xylem)			Adult plants (Secondary xylem)*		
*C. imbricata*	30	70	0.43	30	70	0.4
*E. platyacanthus*	41	59	0.68	40	60	0.7
*F. pilosus*	35	65	0.55	72	22	3.5
*L. lychnidiflora*	42	58	0.72	48/62**	52/38**	0.9/1.6
*L. marginatus*	32	68	0.47	51	49	1.1
*M. carnea*	39	61	0.63	42	58	0.72
*M. geometrizans*	21	79	0.26	38	62	0.6
*O. streptacantha*	31	69	0.45	53/69**	47/31**	1.1/2.2

*Values taken from Reyes-Rivera et al. (2015), except M. carnea, whose values were obtained from samples derived from Maceda et al. (2018) and read by FTIR

**Values taken from Reyes-Rivera et al. (2018).

By comparing syringyl and guaiacyl percentages of the primary xylem with those in the secondary xylem (**Table 5**), it was noticeable that S/G proportion was similar in *C. imbricata*, *E. platyacanthus*, and *M. carnea*. The S/G proportion differs in other species where the percentages of syringyl increased and the percentages of guaiacyl decreased. For example, in *F. pilosus*, primary xylem S/G ratio was 0.55 and in secondary xylem was 3.5, due to the percentage of syringyl that went from 35% to 72%. In the other species studied, syringyl percentages increased between 3% and 22% (**Table 5**).

### Topochemistry of Primary Xylem

**Figure 5:**
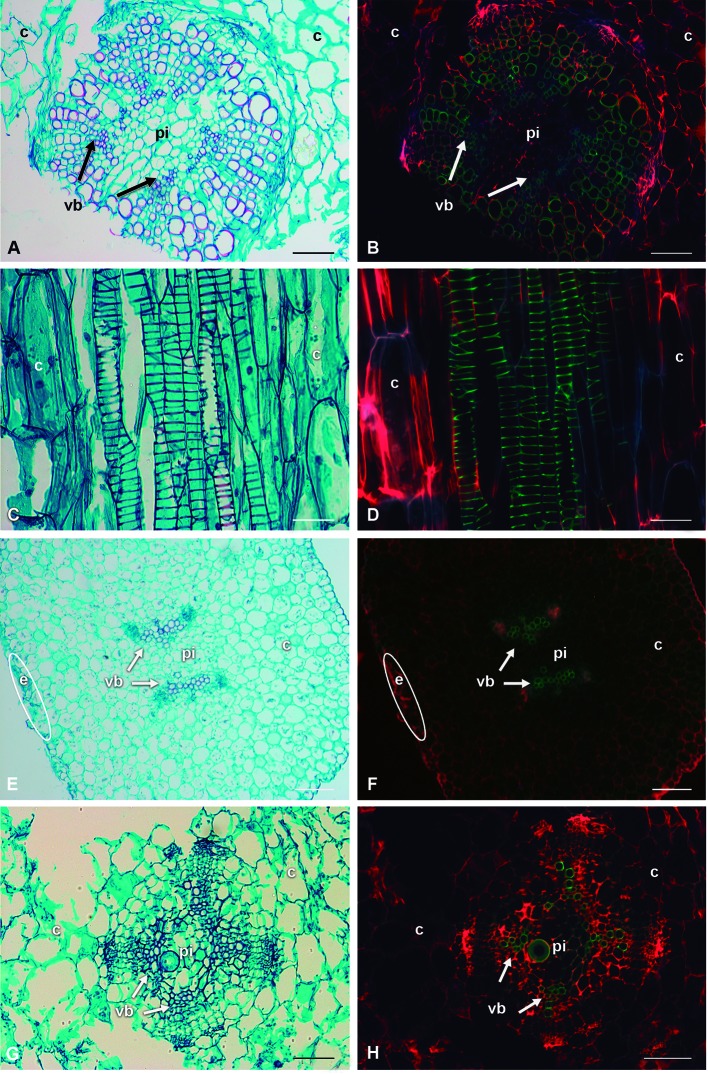
Comparison of bright-field illumination and fluorescence microscopy with triple-band excitation of seedling stems. **(A**, **C**, **E**, **G)** Bright-field illumination. **(B**, **D**, **F**, **H)** Fluorescence emission. **(A**, **B)** *Lophocereus marginatus*, transverse sections. **(C**, **D)** *Lophocereus marginatus*, longitudinal sections. **(E**, **F)** *Leuenbergeria lychnidiflora*, transverse sections. **(G**, **H)** *Opuntia streptacantha,* transverse sections. *Scale*: (**A**, **B**, **E**–**H**; 100 µm. **C**, **D**: 50 µm. c, cortex; e, epidermis; pi, pith; vb, vascular bundle).

Proto- and metaxylem were found in the eight species, and in the 8-month-old seedlings the vascular cambium was visible. Vessel element of the protoxylem showed helical-type secondary walls and their lignin chemical composition differed from metaxylem. The use of safranin O/fast green staining with microscopy fluorescence made it possible to identify differences in the fluorescence emission intensity in the stem of the seedlings (**Figure 5**). With a bright-field microscope, VEs and WBTs had the characteristic red lignin staining while the parenchyma had green cellulose staining (**Figures 5A, C, E, G**). With fluorescence excitation, xylem in the vascular bundles had green tonalities; parenchyma from pith and cortex had just red tonalities as well as the epidermis (**Figures 5B, D, F, H**).

**Figure 6:**
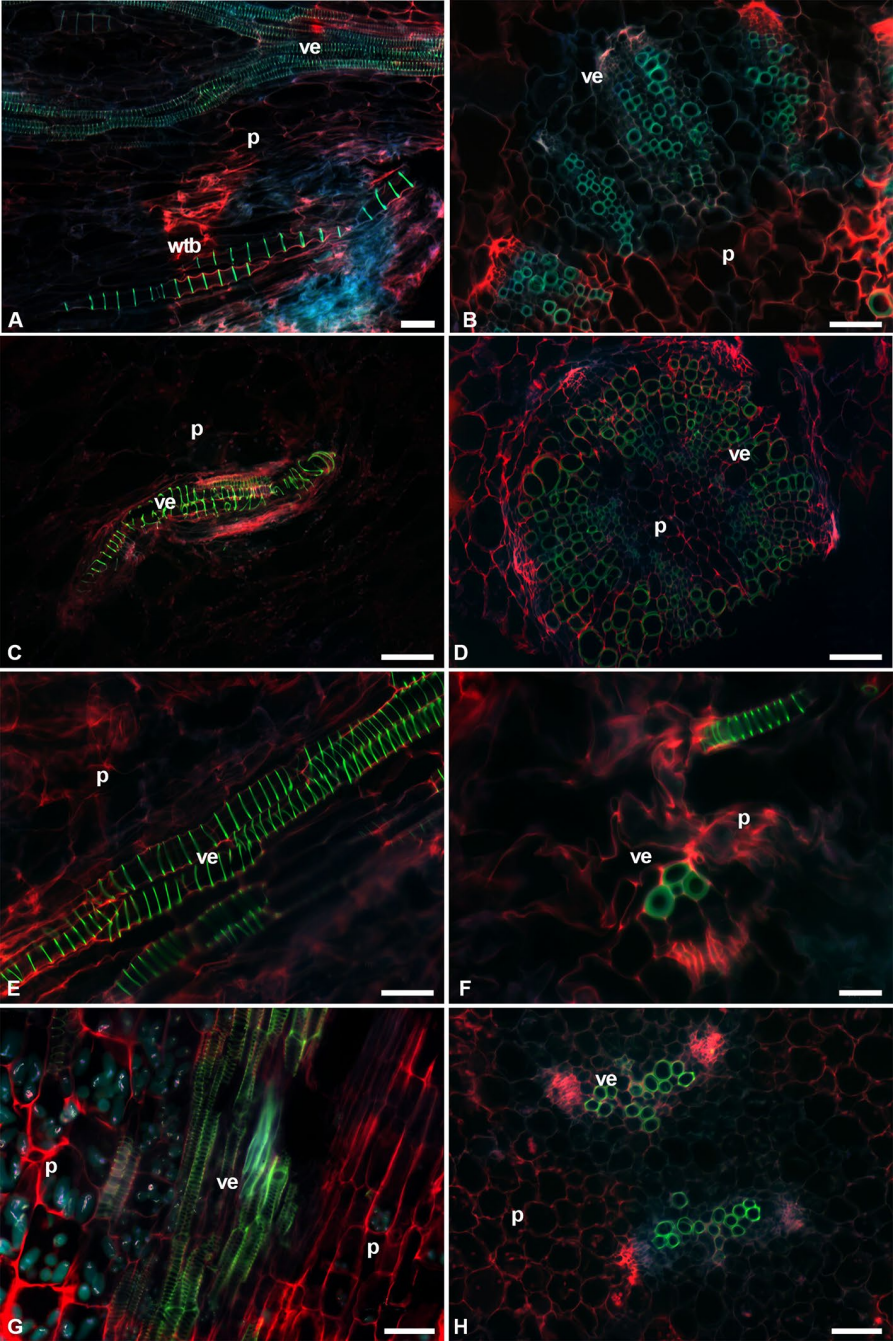
Image seedling stems of fibrous species with triple-band excitation fluorescence. **(A**, **C**, **E**, **G)** Longitudinal sections. **(B**, **D**, **F**, **H**) Transverse sections. **(A**, **B)** *Cylindropuntia imbricata*. **(C**, **D)** *Lophocereus marginatus*. **(E**, **F)** *Myrtillocactus geometrizans*. **(G**, **H)** *Leuenbergeria lychnidiflora*. *Bar* is 50 µm in **(A**, **B**, **D**, **E**, **G**, **H)**; 100 µm in **(C)**; and 20 µm in **(F)**. *p*, parenchyma; *ve*, vessel element; *wbt*, wide-band tracheid.

The fluorescence intensity was lower in the protoxylem with green-blue tones, while in the metaxylem the colors were lime green (**Figure 6**). Most species had VEs of metaxylem with helical-type secondary walls (**Figures 6A, C, E**), but *L. lychnidiflora* had a reticulate-type secondary wall (**Figure 6G**). The fluorescence of lignin in all seedlings had a green to blue emission. The holocellulose and proteins of the middle lamella and part of the primary cell wall were reddish in VEs, WBTs, and parenchyma. The primary xylem was limited by parenchyma cells of the pith. These parenchyma cells were isodiametric, with 10–50 µm in diameter. In the protoxylem of *L. lychnidiflora*, the primary wall of the parenchyma cells was not lignified with abundant starch grains (**Figures 6G, H**). The former can be deduced by fluorescence intensity (green-blue), which is similar to the one found in the VEs of the protoxylem. With the development of metaxylem, the parenchyma in contact to the VEs was narrower.

**Figure 7:**
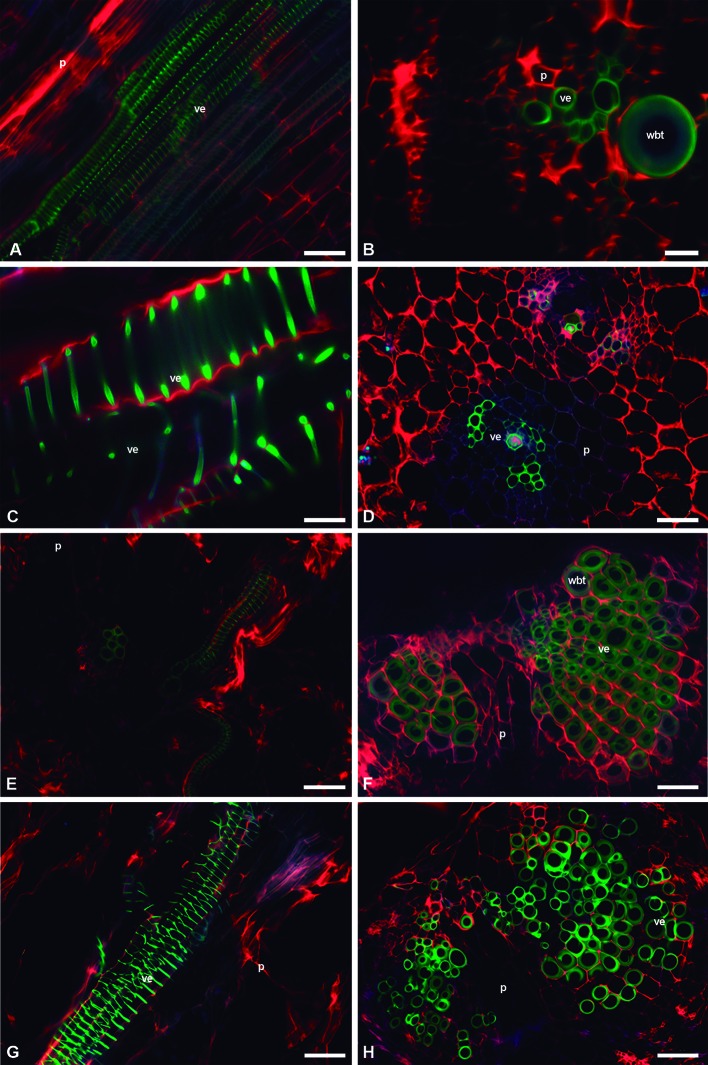
Image seedling stems of fibrous, non-fibrous, and dimorphic species with triple-band excitation fluorescence. **(A**, **C**, **E**, **G)** Longitudinal sections. **(B**, **D**, **F**, **H)** Transverse sections. **(A**, **B)** *Opuntia streptacantha*. **(C**, **D)** *Echinocactus platyacanthus*. **(E**, **F)** *Ferocactus pilosus*. **(G**, **H)** *Mammillaria carnea*. *Bar* is 50 µm in **(A**, **D**–**H)**; 100 µm in **(B)**; and 20 µm in **(C)**. *p*, parenchyma; *ve*, vessel element; *wbt*, wide-band tracheid.

*C*. *imbricata* and *O*. *streptacantha* showed WBTs in the interfascicular region. These WBTs had annular-type secondary walls. The fluorescent emission intensity of lignin in each thickening of the secondary wall in the WBT was weaker than the one found in VEs (**Figures 6A, B** and **7A, B**). The presence of WBTs was scarce in *F. pilosus* and *M. carnea*, but its location was within the vascular bundles. The fluorescent emission intensity of lignin also showed variations between VEs (a higher intensity and lime green coloring) and WBTs (green-blue coloring and a weaker intensity) (**Figures 7E–H**). The parenchyma in *E. platyacanthus* and *M. carnea* had the highest values of size, with a mean of 34.35 and 29.35 µm, respectively (**Figures 7C, D, G, H**), and 23.5 µm in *F. pilosus* (**Figures 7E, F**).

## Discussion

### Chemical Composition

The species that were significantly different in the percentages of seedling extractives and structural components of the cell wall (cellulose and lignin) were the dimorphic ones and the non-fibrous ones compared with the fibrous *L. lychnidiflora* and *L. marginatus*. These results showed that differences exist between the fibrous species and the non-fibrous and dimorphic species since the seedling stage. However, *C. imbricata* and *O. streptacantha*, which are fibrous wood species, did not show significant differences with the dimorphic and non-fibrous wood species during the seedling stage. The lack of differences was probably related to the abundance of parenchyma and the annular- and helical-type secondary walls in VEs and WBTs of both Opuntioideae (**Figures 6A** and **7A**).

When comparing the results of the percentages of seedling extractives with those reported by Maceda et al. (2018) and Reyes-Rivera et al. (2015), a similarity was found between the developmental stages of *C. imbricata*, *F. pilosus*, *L. lychnidiflora*, and *M. carnea*. Moreover, significant differences existed between the four mentioned species with *E. platyacanthus*, *L. marginatus*, *M. geometrizans*, and *O. streptacantha*. The chemical characterization and quantification of the compounds in the extractives is little known. Most of the studies have been focused on the analysis of extractives in alcohol (Reti and Castrillón, 1951; Park et al., 2001). In this study, the use of solvents with different polarities allowed the removal of different extractives. For example, with ethanol/benzene, the phenolic compounds, aldehydes, alcohols, waxes, and some organic acids, were solubilized and extracted (Vargas-Muñoz, 2008; Lansheng et al., 2013; Hernández-Sánchez et al., 2014). With ethanol at 96%, more phenolic and polar compounds were extracted (Carvalho et al., 2012), and with hot water (90°C), starch, gums, and pectin were extracted (Shebani et al., 2009; Campaña et al., 2014).

Cellulose percentages in seedlings were higher than 50% and exceeded those of adult plants (Reyes-Rivera et al., 2015; Maceda et al., 2018). High cellulose percentages were related to the abundance of non-lignified parenchyma and to the helical-type secondary walls in most of the conductive elements, VEs and WBTs; thus, non-lignified primary walls were more abundant (**Figures 6** and **7**). On the contrary, the presence of lignin showed percentages lower than 10% (**Table 3**), while fibrous and dimorphic adult plants had VEs and fibers with cell walls that had accumulated abundant lignin (Reyes-Rivera et al., 2015).

With the quantification method of Li et al. (2017), the total amount of hemicellulose could be obtained, in contrast with other methods that only quantify one type of hemicellulose, such as xylan, for example. In seedlings, hemicellulose percentages were also high; this was mainly due to the abundance of parenchyma in seedlings, which is congruent with the results by Maceda et al. (2018) in non-fibrous species of *Coryphantha*, *Echinocereus*, and *Mammillaria*.

Most of the studies on the chemical structure of primary xylem focused on the lignocellulose compounds because of the importance of forage species in animal nutrition. In the species reported in the literature, lignocellulose compound percentages were different even within the same family (Bidlack and Buxton, 1992; Kamarullah et al., 2015; Ren et al., 2015; **Table S1**). Therefore, the percentages of Cactaceae extractives were similar to some of the species in the Poaceae, possibly because of the accumulation of non-structural carbohydrates and other soluble compounds (Mohammadkhani et al., 1998; Butkuté et al., 2013). Regarding the lignocellulose compounds, only three species showed similar cellulose values to those found in the seedlings of Cactaceae. This means that cacti accumulated a higher quantity of cellulose in their cell walls of the stem, while the other species with primary xylem accumulated lower percentages (Zhang et al., 2015; Ekpo et al., 2016; <47%, **Table S1**).

The percentages of hemicellulose in the Cactaceae seedlings were similar to those reported in the Fabaceae family, which were mainly galactomannans, while in the Poaceae family the percentages of hemicellulose were usually higher (>23%), but of the beta-glucan type (Scheller and Ulvskov, 2010) (**Table S1**). Future research in Cactaceae will focus on the type of hemicellulose present. Regarding lignin, the percentages for *Cannabis sativa* (Cannabaceae) and *Pennisetum purpureum* (Poaceae) were similar to those found in Cactaceae seedlings. It is interesting that in the primary xylem of Cactaceae, the percentages of lignin were closer to those in herbaceous species, in contrast to the reports by Maceda et al. (2018), where the values of the secondary xylem of non-fibrous and some fibrous Cactaceae species were similar to those of hardwood and softwood species (gymnosperms). Only the secondary xylem of *Opuntia* species had similarities with the bamboo *Gigantochloa brang* (**Table S1**) (Maceda et al., 2018).

Therefore, during the seedling stage of Cactaceae, a larger quantity of cellulose was accumulated, while in the development of the secondary xylem, the accumulation of lignin was higher in the secondary cell walls of VEs and fibers, especially in fibrous species, while in non-fibrous species lignin occurred in WBTs and VEs. The results for fibrous wood supported what happens in other groups of woody plants (Novaes et al., 2010; Barros et al., 2015).

The principal component analysis on seedling composition showed that the variable with the highest impact for group separation was the percentage of extractive-free stem (**Table 4**). This was related to what has been reported in the literature, where it was mentioned that during the development of primary xylem, plant biomass increases mainly acropetally, due to procambium activity, originating protoxylem and metaxylem, which can be distinguished anatomically by diameter and lignification types of the secondary wall (Kubo et al., 2005; Růžička et al., 2015). The water percentages at 90°C also had a high contribution in the first component, which would entail that there was a large accumulation of water-soluble compounds that could be related mainly to some compounds such as mucilage (Alalor et al., 2014; Arasi et al., 2016) and non-structural carbohydrates (Plavcová and Jansen, 2015). Because principal component analysis indicated the importance of the extractives in seedlings and adult plants, it is important to quantify and identify each compound present in the different types of extraction in future research.

In the second component, hemicelluloses and celluloses had the greatest importance to separate the species. Unlike what happens in the secondary xylem of cacti, where lignin accumulation was the most important one, cellulose in seedlings acquired a greater relevance because the lignification is a high-cost metabolic process (Amthor, 2003; Barceló et al., 2004; Novaes et al., 2010). During the early development stages of plant growth, there was a larger accumulation of holocellulose in cell walls of parenchyma, VEs and WBTs (Pereira et al., 2018), allowing the elongation to a lower metabolic investment.

During the development of primary xylem, the predominance of helical-type secondary walls in VEs and annular type in WBTs in proto- and metaxylem, as well as reticular type in secondary walls in the metaxylem of *Leuenbergeria*, agrees with the type of cell walls described for many other taxa (Bailey, 1944; Bierhorst and Zamora, 1965; Carlquist, 2009). The limited secondary wall and the high proportion of unlignified primary wall in these tracheary elements (VEs and WBTs) were the major characteristics that allow us to understand why cellulose was the main chemical compound of primary xylem in Cacteae (**Table 4**). Moreover, for secondary xylem, lignin was the most important variable, which contributes to group separation (**Figure 3B**). Species with fibrous wood had VEs with a higher lignin accumulation and pseudoscalariform or alternate pits; in addition to the presence of fibers and lignified axial and radial parenchyma, except for *O. streptacantha* that had unlignified parenchyma. In the case of *M. carnea*—a non-fibrous species—secondary xylem had VEs with mainly helical thickenings embedded in a matrix of WBTs with similar secondary wall thickenings (Reyes-Rivera et al., 2015; Maceda et al., 2018).

During seedling stage, to withstand droughts, cacti bets on the presence of abundant parenchyma cells and mucilage, such as it occurs in non-fibrous species of adult Cactaceae (Maceda et al., 2018). In addition, the occurrence of helical-type secondary walls and the inclination degrees of the helixes allow the VEs to have a higher wettability and efficiency in water ascent (Kohonen and Helland, 2009; McCully et al., 2014). These helical and annular types of secondary wall provide VEs with resistance to endure the tensions generated in the water column by changes in water potential in the seedling. Both types of thickenings of the secondary wall function as supports or rings that “hold” the cell walls joined, preventing them from collapsing or breaking during changes in water potential (Karam, 2005), and represent an adaptive biomechanical advantage to endure drought conditions by allowing VEs and WBTs to bend without lumen closure (Garrett et al., 2010).

This trait of VEs and WBTs with helical thickenings happens in the primary xylem of Cactaceae seedlings and continues during the first year of life of most Cactoideae species and in the adult stage of most Cacteae species (Loza-Cornejo and Terrazas, 2011; Vázquez-Sánchez et al., 2017). However, in the case of *L. lychnidiflora*, VEs change during metaxylem development from a helical- to reticular-type secondary wall, a phenomenon that had not been observed in other species of Cactaceae. Several authors interpret the presence of annular- or helical-type secondary walls as a retention of juvenile traits (Box and Glover, 2010; Dulin and Kirchoff, 2010; Carlquist, 2012). The retention of this type of secondary walls in the water-conductive cells in the adult plants is considered an adaptive trait and happens mainly in small-sized plants, which have to endure adverse conditions especially of hydric stress (Vázquez-Sánchez et al., 2017).

### Topochemistry of Lignin

The innovation of lignin in xylem cell walls allowed the development of tracheary elements and the establishment of plants in various terrestrial environments and diverse ecological niches (Popper et al., 2011; Cosgrove and Jarvis, 2012; Renault et al., 2019). The success in the distribution of angiosperms is due to xylem diversity and lignin (different proportions of lignin S/G) (Sperry et al., 2007; Weng and Chapple, 2010; Feild and Wilson, 2012). The results in the lignin structure of the primary xylem of Cactaceae were consistent among the different species because the guaiacyl monomer predominates in the proto- and metaxylem. In the secondary xylem of most of the adult plants (except for *C. imbricata*, *E. platyacanthus*, and *M. carnea*), the syringyl monomer predominates, as for *F. pilosus* (**Table 5**).

The use of fluorescence microscopy with triple-band excitation and the safranin O/fast green staining allowed to identify the differences in the chemical composition of cell walls from VEs and WBTs mainly, similar to the results of Frank et al. (2007) and Bond et al. (2008) with the same staining and fluorescence microscopy. The bands of wavelengths used were in the range of the reports from different authors: 488–514 nm (Speranza et al., 2009), 450–490 nm (Bergau et al., 2016), and 488–568 nm (Bond et al., 2008), with sequential excitation (355, 488, and 633 nm) or two bands at the same time (488 and 561 nm) (Donaldson and Williams, 2018), and especially with the bands used by Angeles et al. (2004) who used the same fluorescence emission band for lignin (505–530) as the band used in this work. Therefore, with the three bands of excitation (385–400, 475–490, and 545–565), the blue and green characteristic tonalities of lignin were observed (Albinsson et al., 1999; Radotić et al., 2006; Bond et al., 2008; Speranza et al., 2009; Donaldson, 2013). In addition, fluorescence intensity reflects the concentration of lignin, with higher intensity representing a higher accumulation of lignin (Ji et al., 2013) (**Figure 5**).

**Figure 8:**
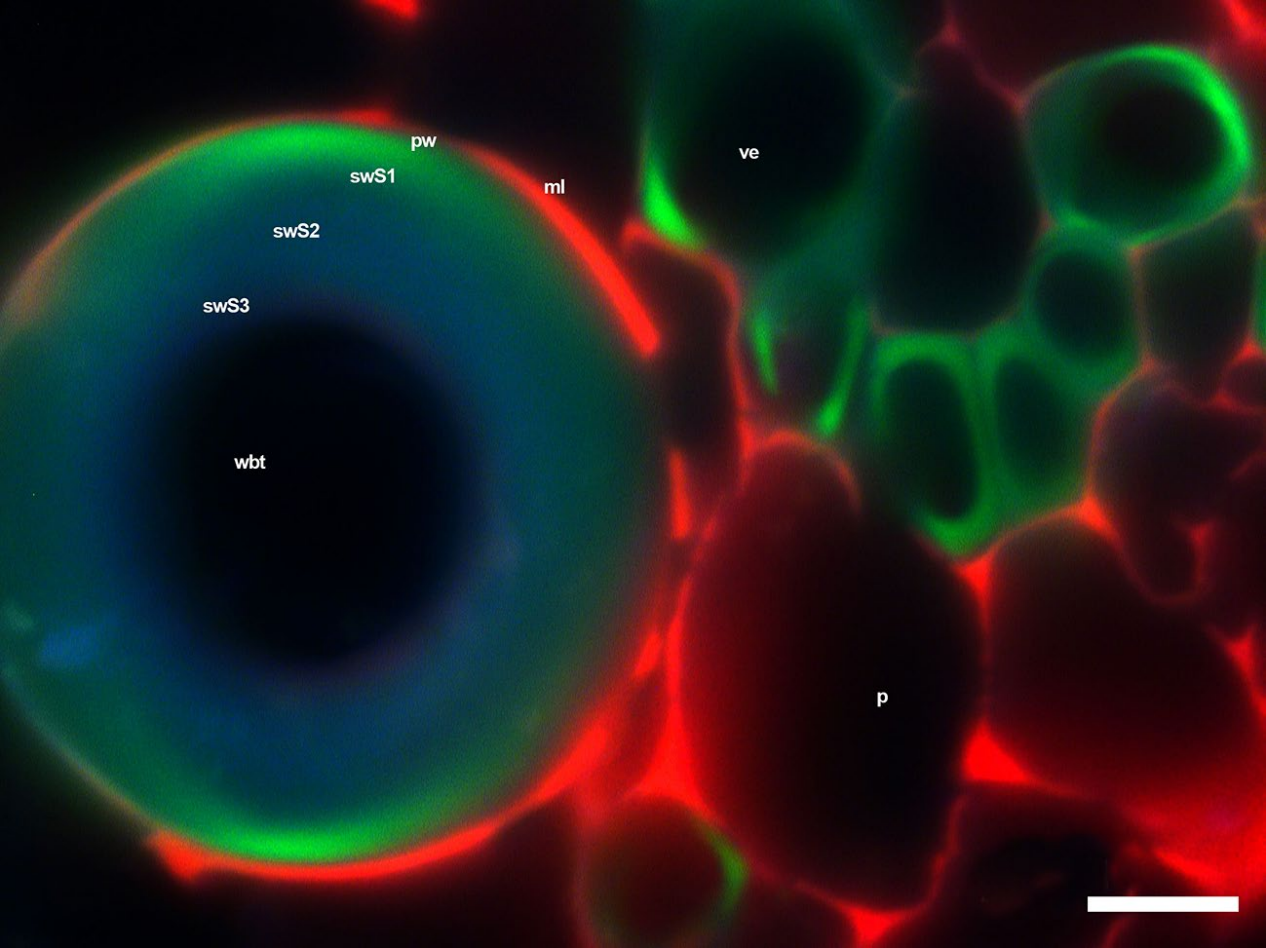
WBTs and VEs of *Opuntia streptacantha*, where differences in fluorescence intensity were observed with the triple-band excitation. Layers 2 and 3 of the secondary wall have a green-blue color, while part of layer 1 of the secondary wall has a lime green color, similar to the one found in the secondary wall thickenings in VEs. *Bar* is 10 µm. *p*, parenchyma; *pw*, primary wall; *swS1*, first layer of secondary wall; *swS2*, second layer of secondary wall; *swS3*, third layer of secondary wall 3; *ml*, middle lamella; *ve*, vessel element; *wbt*, wide-band tracheid.

The fluorescence observations of the primary xylem in the seedlings showed that VEs had a higher fluorescence intensity, while WBT’s intensity was higher in the S1 layer of the secondary wall than in the S2 and S3 layers of the same wall (**Figure 8**). This would explain the values shown in **Table 5** since syringyl levels were lower than those for guaiacyl because of a predominance of VE, whose composition was mainly G-type lignin (Zhou et al., 2011a; Saito et al., 2012), while WBTs accumulated mainly S-type lignin, but the G-type in the S1 layer of the secondary wall was also observed (**Figure 8**). The predominance of guaiacyl in the primary xylem was in agreement with that reported by Li et al. (2001) for angiosperms. The middle lamella and part of the primary cell wall in VEs, WBTs, and parenchyma showed a red intense color due to holocellulose (Donaldson et al., 1999; Donaldson and Knox, 2012) and proteins or enzymes (Donaldson and Vaidya, 2017).

Comparisons of syringyl and guaiacyl percentages in the primary xylem with those in secondary xylem reported by Reyes-Rivera et al. (2015; 2018) indicated that syringyl percentages were much higher in adult plants than those present in seedlings. This is possibly due to the fact that some species in the adult stage have fibers and the cell wall of VEs are completely lignified with pseudoscalariform pits, which influence syringyl and guaiacyl percentages. Moreover, fibers have a higher syringyl percentage (Obst and Ralph, 1983; Takabe et al., 1992; Watanabe et al., 2004). In the case of the non-fibrous species *M. carnea*, guaiacyl proportion was very similar in seedlings and in adult plants due to the presence of VEs and WBTs with annular- and helical-type secondary walls both in the primary (**Figure 7**) and secondary xylem (Maceda et al., 2018). The occurrence of these tracheary elements and their chemical composition could be related to the paedomorphosis process reported for Cactaceae (Altesor et al., 1994; Mauseth, 2004; Dulin and Kirchoff, 2010).

The accumulation of syringyl-type lignin can vary within the same plant due to stressing environmental factors (Donaldson, 2002; Lourenço et al., 2016; Malavasi et al., 2016), mainly between early and latewood, where syringyl accumulation is higher in earlywood, while in latewood the syringyl percentage is lower and the guaiacyl percentage is higher. According to various authors, this change in lignin type allows drought conditions to be better tolerated (Takabe et al., 1992; Watanabe et al., 2004; Saito et al., 2012). Thus, the presence of a high quantity of guaiacyl in the seedlings of Cactaceae would be similar to what occurs in latewood of secondary xylem, which would give VEs in primary xylem a higher resistance to drought conditions.

The layered accumulation of the secondary wall observed in the WBTs in **Figure 8** was similar to what has been reported for other species (Prislan et al., 2009; Zeng et al., 2015). Guaiacyl accumulated in the primary wall and the first layer of secondary wall (swS1), and later in the second layer (swS2) (Grünwald et al., 2002; Boyce et al., 2004; Grabber, 2005; Barros et al., 2015), where syringyl monomers also accumulated in a gradual manner (Yoshizawa et al., 1999; Donaldson, 2001; Donaldson, 2013). The presence of guaiacyl in the primary wall and S1 layer of the secondary wall in WBTs and VEs provides high resistance to compression and also gives more rigidity and support to VEs (Yoshizawa et al., 1993a and Yoshizawa et al., 1993b; Donaldson, 2001; Barceló et al., 2004).

The presence of syringyl lignin in the inner layers of the secondary wall of the WBTs could occur because lignin with syringyl/guaiacyl is less condensed and more hydrophilic, which allows water to flow in a more efficient way (Pereira et al., 2018). Thus, efficiency in water transport would allow the seedling to efficiently prevent the presence of embolisms and to avoid cavitation of VEs by allowing the flow of water through the WBT lumen and later through the non-lignified primary wall into the adjacent VE, as it has been suggested by several authors (Mauseth et al., 1995; Landrum 2006). Therefore, differences in lignin composition in Cactaceae seedlings, along with other factors such as succulence (Linton and Nobel, 2001; Nerd and Neumann, 2004), WBT presence (Loza-Cornejo et al., 2003; Mauseth, 2004; Landrum, 2006), and helical-type secondary walls in VEs (Karam, 2005), would allow to better endure drought conditions during their early stages by accumulating more guaiacyl-type lignin. This situation is modified during the development of the secondary xylem in most species of Cactaceae, where syringyl-type lignin prevails in the tracheary elements, not only as a structural component (Reyes-Rivera et al., 2015; Reyes-Rivera et al., 2018) but also as a protection against oxidative agents of fungi (Menden et al., 2007; Skyba et al., 2013) or other pathogenic organisms (Martone et al., 2009). The use of other complementary methods such as time-of-flight secondary ion mass spectrometry (TOF-SIMS) (Saito et al., 2012), UV microspectrophotometry (Koch, 2004), along with precise measuring techniques like NMR and Py-GC/MS (Reyes-Rivera et al., 2018), will allow for a better understanding of the structural chemical composition of the primary xylem of Cactaceae species.

## Conclusion

The principal components of the primary xylem of Cactaceae were extractive-free stem, water-soluble compounds, and holocellulose, which foster quick growth at a low energetic expense. The retention of annular- and helical-type secondary walls in the secondary xylem, along with the similarity in the type of lignin present in the primary and secondary xylem of *M. carnea*, supported the paedomorphism for this non-fibrous species of Cactaceae, but not for the other species.

The efficiency in the accumulation and ascent of water was due to the presence of abundant parenchyma cells and annular- and helical-type secondary walls in VEs and WBTs. The syringyl-type lignin in the S2 and S3 layers of the secondary walls of WBTs makes the flow of water easier and prevents embolism and cavitation in VEs, whereas the guaiacyl lignin in the primary and secondary walls (S1) contributes to the rigidity and support of WBTs. The resistance and flexibility of VEs occur because of the homogeneous accumulation of guaiacyl lignin in the cell wall and its helical thickenings.

The use of different techniques and methods in the study of the anatomy and the structural chemical composition of tracheary elements, both in the primary and secondary xylem, has allowed us to further understand the adaptations and strategies that Cactaceae species use to survive and adapt to the different conditions of the environments where they live and thrive.

## Data Availability Statement

The raw data supporting the conclusions of this manuscript will be made available by the authors, without undue reservation, to any qualified researcher. Figshare doi: 10.6084/m9.figshare.9776075

## Author Contributions

AM and TT designed the work. AM performed the experiments and prepared the figures. AM, TT, MS-H, CP-V, and CT analyzed the data. AM and TT wrote the manuscript. All the authors have read and approved the manuscript.

## Conflict of Interest

The authors declare that the research was conducted in the absence of any commercial or financial relationships that could be construed as a potential conflict of interest.

## References

[B1] Abreu C. L. R. Orea-Igarza U. Cordero-Manchado E. (2004). Composición química de tres maderas en la provincia de Pinar del Río, Cuba a tres alturas del fuste comercial. Parte No. 1: *Corymbia citriodora* . Rev. Chapingo. Serie Cienc. Forestales y del Ambiente. 10, 57–62.

[B2] Alalor C. A. Avbunudiogba J. A. Augustine K. (2014). Isolation and characterization of mucilage obtained from *Colocasia esculenta* . Int. J. Pharm. Biol. Sci. 4, 25–29. 10.1002/9781119441632.ch9

[B3] Albinsson B. Li S. Lundquist K. Stomberg R. (1999). The origin of lignin fluorescence. J. Mol. Struct. 508, 19–27. 10.1016/S0022-2860(98)00913-2

[B4] Altesor A. Ezcurra E. (2003). Functional morphology and evolution of stem succulence in cacti. J. Arid Environ. 53, 557–567. 10.1006/jare.2002.1059

[B5] Altesor A. Silva C. Ezcurra E. (1994). Allometric neoteny and the evolution of succulence in cacti. Bot. J. Linn. Soc. 114, 283–292. 10.1006/bojl.1994.1018

[B6] Amthor J. S. (2003). Efficiency of lignin biosynthesis: a quantitative analysis. Ann. Bot. 91, 673–695. 10.1093/aob/mcg073 12714366 PMC4242356

[B7] Angeles G. Owens S. A. Ewers F. W. (2004). Fluorescence shell: a novel view of sclereid morphology with the confocal laser scanning microscope. Microsc. Res. Techniq. 63, 282–288. 10.1002/jemt.20043 15170758

[B8] Arasi M. A. S. A. G. Rao M. G. Bagyalakshmi J. (2016). The comparison and analysis of two extraction methods for polysaccharides in *Psidium guajava* L. fruits. Indian J. Pharm. Educ. Res. 50, 218–224. 10.5530/ijper.50.3.32

[B9] Arnold D. H. Mauseth J. D. (1999). Effects of environmental factor on development of wood. Am. J. Bot. 86, 367–371. 10.2307/2656758 10077499

[B10] Arruda E. Melo de Pinna G. F. (2010). Wide-band tracheids (WBTs) of photosynthetic and non-photosynthetic stems in species of Cactaceae. J. Torrey. Bot. Soc 137, 16–29. 10.3159/09-RA-052.1

[B11] Bárcenas-Argüello M. L. López-Mata L. Terrazas T. García-Moya E. (2013). Germinación de tres especies de *Cephalocereus* Cactaceae endémicas del Istmo de Tehuantepec, México. Polibotánica. 36, 105–116.

[B12] Bailey I. W. (1944). The development of vessels in angiosperms and its significance in morphological research. Am. J. Bot. 31, 421–428. 10.1002/j.1537-2197.1944.tb08053.x

[B13] Barceló A. R. Gómez-Ros L. V. Gabaldón C. López-Serrano M. Pomar F. Carrión J. S. (2004). Basic peroxidases: the gateway for lignin evolution?. Phytochem. Rev. 3, 61–78. 10.1023/B:PHYT.0000047803.49815.1a

[B14] Barros J. Serk H. Granlund I. Pesquet E. (2015). The cell biology of lignification in higher plants. Ann. Bot. 115, 1053–1074. 10.1093/aob/mcv046 25878140 PMC4648457

[B15] Baucher M. El Jaziri M. Vandeputte O. (2007). From primary to secondary growth: origin and development of the vascular system. J. Exp. Bot. 58, 3485–3501. 10.1093/jxb/erm185 17898423

[B16] Bergau N. Santos A. N. Henning A. Balcke G. U. Tissier A. (2016). Autofluorescence as a signal to sort developing glansular trichomes by flow cytometry. Front. Plant Sci. 7, 949. 10.3389/fpls.2016.00949 27446176 PMC4923063

[B17] Bevilaqua M. Filho A. P. S. Mangolin C. A. Oliveira A. J. B. Machado M. F. P. S. (2015). Genetic and chemical diversity in seeds of cactus mandacaru (*Cereus* sp.) from two edaphoclimatic regions contrasting. An. Acad. Bras. Ciênc. 87, 765–776. 10.1590/0001-3765201520140029 26131634

[B18] Bidlack J. E. Buxton D. R. (1992). Content and deposition rates of cellulose, hemicellulose, and lignin during regrowth of forage grasses and legumes. Can. J. Plant Sci. 72, 809–818. 10.4141/cjps92-097

[B19] Bierhorst D. W. Zamora P. M. (1965). Primary xylem elements and element associations of angiosperms. Am. J. Bot. 52, 657–710. 10.1002/j.1537-2197.1965.tb07236.x

[B20] Bond J. Donaldson L. Hill S. Hitchcock K. (2008). Safranine fluorescent staining of wood cell walls. Biotech. Histochem. 83, 161–171. 10.1080/10520290802373354 18802812

[B21] Box M. S. Glover B. J. (2010). A plant developmentalist’s guide to paedomorphosis: reintroducing a classic concept to a new generation. Trends Plant Sci. 15, 241–246. 10.1016/j.tplants.2010.02.004 20226719

[B22] Boyce C. K. Zwienleckl M. A. Cody G. D. Jacobsen C. Wirick S. Knoll A. H. (2004). Evolution of xylem lignification and hydrogel transport regulation. Proc. Natl. Acad. Sci. U.S.A. 101, 17555–17558. 10.1073/pnas.0408024101 15574502 PMC536047

[B23] Brodersen C. R. McElrone A. J. (2013). Maintenance of xylem network transport capacity: a review of embolism repair in vascular plants. Front. Plant Sci. 4, 108. 10.3389/fpls.2013.00108 23630539 PMC3633935

[B24] Butkuté B. Lemeziené N. Ceseviciené J. Liatukas Z. Dabkeviciené G. (2013). Carbohydrate and lignin partitioning in switchgrass (*Panicum virgatum* L.) biomass as a bioenergy feedstock. ZEMDIRBYSTE 100, 251–260. 10.13080/z-a.2013.100.032

[B25] Campaña. M. L. Tijero A. Aguado R. López M. M. Moral A. (2014). Biorrefinería de residuos de madera. Obtención de celulosa de alta pureza. Biosaia 3, 1–3. 10.13140/2.1.4152.3200

[B26] Cao S. Ma X. Lin L. Huang F. Huang L. Chen L. (2014). Morphological and chemical characterization of green bamboo (*Dendrocalamus oldhamii* (Munro) Keng f.) for dissolving pulp production. BioResources. 9, 4528–4539. 10.15376/biores.9.3.4528-4539

[B27] Carlquist S. Schneider E. L. (2010). Origins and nature of vessels in monocotyledons. 11. Primary xylem microstructure, with examples from Zingiberales. Int. J. Plant Sci. 171, 258–266. 10.1086/650160

[B28] Carlquist S. (2009). Xylem heterochrony: an unappreciated key to angiosperm origin and diversification. Bot. J. Linn. Soc. 161, 26–65. 10.1111/j.1095-8339.2009.00991.x

[B29] Carlquist S. (2012). How wood evolves: a new synthesis. Botany 90, 901–940. 10.1139/b2012-048

[B30] Carvalho R. H. R. Galväo E. L. Barros A. C. Conceicao M. M. Sousa E. M. B. D. (2012). Extraction, fatty acid profile and antioxidant activity of sesame extract (Sesamum indicum L.). Braz. J. Chem. Eng. 29, 409–420. 10.1590/S0104-66322012000200020

[B31] Chang W. J. Chang M. J. Chang S. T. Yeh T. F. (2013). Chemical composition and immunohistological variations of growing bamboo shoot. J. Wood Chem. Technol. 33, 144–155. 10.1080/02773813.2013.769114

[B32] Chaves A. V. Waghorn G. C. Tavendale M. H. (2002). A simplified method for lignin measurement in a range of forage species. Proc. New Z. Grass Assoc. 64, 129–133. 10.1111/gfs.12293

[B33] Cherney J. H. Johnson K. D. Volenec J. J. Anliker K. S. (1988). Chemical composition of herbaceous grass and legume species grown for maximum biomass production. Biomass. 17, 215–238. 10.1016/0144-4565(88)90105-9

[B34] Cosgrove D. J. Jarvis M. C. (2012). Comparative structure and biomechanics of plant primary and secondary cell walls. Front. Plant Sci. 3, 204. 10.3389/fpls.2012.00204 22936943 PMC3424969

[B35] Dürrenberger M. B. Handschin S. Conde-Petit B. Escher F. (2001). Visualization of food structure by confocal laser scanning microscopy (CLSM). LWT-Food Sci. Technol. 34, 11–17. 10.1006/fstl.2000.0739

[B36] Danzer S. R. Leavitt S. W. Panyushkina I. P. Mergner A. Garcia E. Best-Svob V. (2001). Xylem tracheid development in *Pinus resinosa* seedlings in controlled environments. Tree Ring Res. 57, 45–53.

[B37] De Micco V. Aronne G. (2007). Combined histochemistry and autofluorescence for identifying lignin distribution in cell walls. Biotech. Histochem. 82, 209–216. 10.1080/10520290701713981 18074267

[B38] Decou R. Serk H. Ménard D. Pesquet E. (2017). “Analysis of lignin composition and distribution using fluorescence laser confocal microspectroscopy, in Xylem: methods and protocols, methods in molecular biology. Eds. De-Lucas J. P. Etchells M. (Durham: Human Press), 233–247. 10.1007/978-1-4939-6722-3_17 28050840

[B39] Dence C. W. (1992). “The determination of Lignin” in Methods in lignin chemistry. Eds. Lin C. W. Dence S. Y. (Syracuse: College of Environmental Science and Forestry), 3–61.

[B40] Dinno A. (2015). Nonparametric pairwise multiple comparisons in independent groups using Dunn’s test. Stata J. 15, 292–300. 10.1177/1536867X1501500117

[B41] Donaldson L. Knox J. P. (2012). Localization of cell wall polusaccharides in normal and compression wood of radiate pine: relationships with lignification and microfibril orientation. Plant Physiol. 158, 642–653. 10.1104/pp.111.184036 22147521 PMC3271756

[B42] Donaldson L. Vaidya A. (2017). Visualizing recalcitrance by colocalisation of cellulose, lignin and cellulose in pretreated pine biomass using fluorescence microscopy. Sci. Rep. 7, 1–13. 10.1038/srep44386 28281670 PMC5345003

[B43] Donaldson L. Williams N. (2018). Imaging spectroscopy of natural fluorophores in Pine needles. Plants. 7, 1–16. 10.3390/plants7010010 PMC5874599 29393922

[B44] Donaldson L. A. Singh A. P. Yoshinaga A. Takabe K. (1999). Lignin distribution in mild compression wood of *Pinus radiata* . Can. J. Bot. 77, 41–50. 10.1139/b98-190

[B45] Donaldson L. Radotić K. Kalauzi A. Djikanović D. Jeremić M. (2010). Quiantification of compression wood severity in tracheids of *Pinus radiate* D. Don using confocal fluorescence imagins and spectral deconvolution. J. Struct. Biol. 169, 106–115. 10.1016/j.jsb.2009.09.006 19747548

[B46] Donaldson L. A. (2001). Lignification and lignin topochemistry – an ultraestructural view. Phytochemistry 54, 859–873. 10.1016/s0031-9422(01)00049-8 11423137

[B47] Donaldson L. A. (2002). Abnormal lignin distribution in wood from severely drougth stressed *Pinus radiata* trees. IAWA J. 23, 161–178. 10.1163/22941932-90000295

[B48] Donaldson L. (2013). Softwood and hardwood lignin fluorescence spectra of wood cell walls in different mounting media. IAWA J. 34, 3–19. 10.1163/22941932-00000002

[B49] Dulin M. W. Kirchoff B. K. (2010). Paedomorphosis, secondary woodiness, and insular woodiness in plants. Bot. Rev. 76, 405–490. 10.1007/s12229-010-9057-5

[B50] Ekebafe L. O. Ekebafe M. O. Akpa F. A. O. Erhuanga G. Etiobhio B. W. (2011). Graft copolymerization of acrylonitrile onto delignified native bamboo (*Bambusa vulgaris*) cellulosic and its utilization potential for heavy metal uptake from aqueous medium. Chem. Ind. Chem. Eng. Q. 17, 133–140. 10.2298/CICEQ101021063E

[B51] Ekpo I. Ogali R. Ofodile S. Achugasim O. (2016). Comparison of biomass content of the evaluation of cellulosic ethanol fuel production from predominant perennial grasses in South-South, Nigeria. IJAST 6, 38–46. 10.2134/agronj2016.08.0454

[B52] Feild T. S. Wilson J. P. (2012). Evolutionary voyage of Angiosperm vessel structure-function and its significance for early Angiosperm success. Int. J. Plant Sci. 173, 596–609. 10.1086/666099

[B53] Feild T. S. Zweiniecki M. A. Brodribb T. Jaffré T. Donoghue M. J. Holbrook M. (2000). Structure and function of tracheary elements in *Amborella trichopoda* . Int. J. Plant Sci. 161, 705–712. 10.1086/314293

[B54] Fonseca-Prieto F. Canché-Escamilla G. Chavarria-Hernández J. C. Duarte-Aranda S. (2014). Characterization of lignocellulosic residues of henequen and their use as a bio-oil source. Biomass Convers. Biorefin. 4, 95–104. 10.1007/s13399-013-0099-x

[B55] Frank J. H. Elder A. D. Swartling J. Venkitaraman A. R. Jeyasekharan A. D. Kaminski C. F. (2007). A white light confocal microscope for spectrally resolved multidimensional imaging. J. Microsc. 227, 203–215. 10.1111/j.1365-2818.2007.01803.x 17760615

[B56] Garrett T. Y. Huynh C. V. North G. B. (2010). Root contraction helps protect the “living rock” cactus *Ariocarpus fissuratus* from lethal high temperatures when growing in rocky soil. Am. J. Bot. 97, 1951–1960. 10.3732/ajb.1000286 21616844

[B57] Gibson A. C. (1978). Woody anatomy of Platyopuntias. Aliso 9, 279–307. 10.5642/aliso.19780902.08

[B58] Grünwald C. Ruel K. Schmitt U. (2002). Differentiation of xylem cells in *rol*C transgenic aspen trees – a study of secondary cell wall development. Ann. For. Sci. 59, 679–685. 10.1051/forest:2002056

[B59] Grabber J. H. (2005). How do lignin composition, structure, and cross-linking affect degradability? A review of cell wall model studies. Crop Sci. 45, 820–831. 10.2135/cropsci2004.0191

[B60] Guillén S. Terrazas T. De la Barrera E. Casas A. (2011). Germination differentiation patterns of wild and domesticated columnar cacti in a gradient of artificial selection intensity. Genet. Resour. Crop Evol. 58, 409–423. 10.1007/s10722-010-9586-0

[B61] Han J. Rowell J. (1996). “Chemical composition of fibers”, in Paper and composites from agro-based, vol. 83 Eds. Rowell R. A. Young J. K. Rowell R. M. (US: Lewis Publishers), 134.

[B62] Haseloff J. (2003). Old botanical techniques for new microscopes. Biotechniques 34, 1174–1182. 10.2144/03346bi01 12813885

[B63] Hernández-Sánchez R. Lami-Izquierdo L. Pino-Olea J. Cámara-Pérez A. Martínez-Pérez Y. (2014). Identificación básica de extractivos en alcohol benceno del bagazo de la caña de azúcar mediante CG/EM. ICIDCA 48, 16–20.

[B64] Huang B. Q. Yeung E. C. (2015). Chemical and physical fixation of cell and tissues: an overview, in Plant microtechniques and protocols. Ed. Yeung (Cham: Springer). 23–43. 10.1007/978-3-319-19944-3_2

[B65] Jääskeläinen A.-S. Nuopponen M. Axelsson P. Tenhunen M. Löija M. Vuorinen T. (2003). Determination of lignin distribution in pulps by FTIR ATR Spectroscopy. J. Pulp Pap. Sci. 29, 328–331.

[B66] Jaramillo-Carmona S. Fuentes-Alventosa J. M. Rodríguez-Gutiérrez G. Waldron K. W. Smith A. C. Guillén-Bejarano R. (2008). Characterization of Asparagus lignin by HPLC. J. Food Sci. 73, 526–532. 10.1111/j.1750-3841.2008.00893.x 18803697

[B67] Ji Z. Ma J.-F. Zhang Z.-H. Xu F. Sun R.-C. (2013). Distribution of lignin and cellulose in compression wood tracheids of *Pinus yunnanensis* determined by fluorescence microscopy and confocal Raman microscopy. Ind. Crop Prod. 47, 212–217. 10.1016/j.indcrop.2013.03.006

[B68] Jin Z. Shao S. Katsumata K. S. (2007). Lignin characteristics of peculiar vascular plants. J. Wood Sci. 53, 520–523. 10.1007/s10086-007-0891-y

[B69] Jung H. G. Vogel K. P. (1986). Influence of lignin on digestibility of forage cell wall material. J. Anim. Sci. 62, 1703–1712. 10.2527/jas1986.6261703x 3733564

[B70] Kamarullah S. H. Mydin M. M. Omar W. S. W. Harith S. S. Noor B. H. M. Alias N. Z. A. (2015). Surface morphology and chemical composition of napier grass fibers. MJAS 19, 889–895.

[B71] Karam G. N. (2005). Biomechanical model of the xylem vessels in vascular plants. Ann. Bot. 95, 1179–1186. 10.1093/aob/mci130 15802309 PMC4246902

[B72] Kiyoto S. Yoshinaga A. Fernandez-Tendero E. Day A. Chabbert B. Takabe K. (2018). Distribution of lignin, hemicellulose, and arabinogalactan protein in hemp phloem fibers. Microsc. Microanal. 24, 442–452. 10.1017/S1431927618012448 30175708

[B73] Klap V. A. Hemminga M. A. Boon J. J. (2000). Retention of lignin seagrasses: angiosperms that returned to the sea. Mar. Ecol. Prog. Ser. 194, 1–11. 10.3354/meps194001

[B74] Kline L. M. Hayes D. G. Womac A. R. Labbé N. (2010). Simplified determination of lignin content in hard and soft woods via UV-Spectrophotometric analysis of biomass dissolved in ionic liquids. BioResources 5, 1366–1383.

[B75] Koch G. (2004). Topochemical characterization of lignins and phenolic extractives in wood cell walls. Lenzinger Ber. 83, 6–12. 10.1515/HF.2003.051

[B76] Kohl K. D. Miller A. W. Dearing M. D. (2014). Evolutionary irony: evidence that ‘defensive’ plant spines act as a proximate cue to attract a mammalian herbivore. Oikos 14, 835–841. 10.1111/oik.02004 PMC8046181 33859445

[B77] Kohonen M. M. Helland A. (2009). On the function of wall sculpturin in xylem conduits. J. Bionic. Eng. 6, 324–329. 10.1016/S1672-6529(08)60131-6

[B78] Kubo M. Udagawa M. Nishikubo N. Horiguchi G. Yamaguchi M. Ito J,. (2005). Transcription switches for protoxylem and metaxylem vessel formation. Genes. Dev. 19, 1855–1860. 10.1101/gad.1331305 16103214 PMC1186185

[B79] Kutscha N. P. McOrmond R. R. (1972). The suitability of using fluorescence microscopy for studying lignification in Balsam Fir. Tech. Bull. Life Sci. Agr. Exp. Sta. Univ. Maine. 62, 15.

[B80] Landrum J. V. (2006). Wide-band tracheids in genera of Portulacaceae: novel, non xylary tracheids possibly evolved as an adaptation to water stress. J. Plant Res. 119, 497–504. 10.1007/s10265-006-0013-8 16896531

[B81] Lansheng W. Wanxi P. Zhi L. Yueping T. Liwen S. (2013). Analysis on molecular characteristics of wood extractives from *Eucalyptus urophydis* biomass. Biotechnol. Indian J. 7, 559–564.

[B82] Latorraca J. V. F. Dünisch O. Koch G. (2011). Chemical composition and natural durability of juvenile and mature heartwood of *Robinia pseudoacacia* L. Anais da Academia Bras. Ciências. 83, 1059–1068. 10.1590/S0001-37652011005000016 21779654

[B83] Li L. Cheng X. F. Leshkevich J. Umezawa T. Harding S. A. Chiang V. L. (2001). The last step of syringyl monolignol biosynthesis in Angiosperms is regulated by a novel gene encoding sinapyl alcohol dehydrogenase. Plant Cell. 13, 1567–1585. 10.1105/TPC.010111 11449052 PMC139549

[B84] Li X. Sun C. Zhou B. He Y. (2015). Determination of hemicellulose, cellulose and lignin in Moso bamboo by near infrared spectroscopy. Sci. Rep. 5, 1–11. 10.1038/srep17210 PMC4658639 26601657

[B85] Li R. Yang G. Chen J. He M. (2017). The characterization of hemicellulose extract from corn stalk with stepwise alkali extraction. J. Korea TAPPI 49, 29–40. 10.7584/JKTAPPI.2017.08.49.4.29

[B86] Linton M. Nobel P. S. (2001). Hydraulic conductivity, xylem cavitation, and water potential for succulent leaves of *Agave deserti* and *Agave tequilana* . J. Plant Sci. 162, 747–754. 10.1086/320782

[B87] Lourenço A. Rencoret J. Chemetova C. Gominho J. Gutiérrez A. del Río J. C. (2016). Lignin composition and structure differs between xylem, phloem and phellem in *Quercus suber* L. Front. Plant Sci. 7, 1612. 10.3389/fpls.2016.01612 27833631 PMC5081372

[B88] Loza-Cornejo S. Terrazas T. (1996). Anatomía del tallo y de la raíz de dos especies de *Wilcoxia* Britton & Rose (Cactaceae) del noreste de México. Bol. Soc. Bot. México. 59, 13–23. 10.17129/botsci.1502

[B89] Loza-Cornejo S. Terrazas T. (2011). Morfo-anatomía de plántulas en especies de Pachycereeae: ¿Hasta cuándo son plántulas? Bol. Soc Bot. México. 88, 1–13. 10.17129/botsci.293

[B90] Loza-Cornejo S. Terrazas T. López-Mata L. Trejo C. (2003). Características morfo-anatómicas y metabolismo fotosintético en plántulas de *Stenocereus queretaroensis* (Cactaceae): Su significado adaptativo. Interciencia. 28 (2), 83–89.

[B91] Loza-Cornejo S. López-Mata L. Terrazas T. (2008). Morphological seed traits and germination of six species of Pachycereeae Cactaceae. J. Prof. Assoc. Cactus. 10, 71–84. 10.1007/s10265-004-0156-4

[B92] Loza-Cornejo S. Aparicio-Fernández X. Patakfalvi R. J. Rosas-Saito G. H. (2017). Caracteres anatómicos y fitoquímicos del tallo y raíz de *Mammillaria uncinata* (Cactaceae). Acta Bot. Mex. 120, 21–38. 10.21829/abm120.2017.1159

[B93] Lucas W. J. Groover A. Lichtenberg R. Futura K. Yadav S. R. Helariutta Y. (2013). The plant vascular system: evolution, development and functions. J. Integr. Plant Biol. 55, 294–388. 10.1111/jipb.12041 23462277

[B94] Luna M. L. Ramos-Giacosa J. P. Giudice G. E. Fernández P. V. Ciancia M. Saparrat M. C. N. (2015). Structure and chemistry of the xylem of arborescent species of *Blechnum* from South America. IAWA J. 36, 3–21. 10.1163/22941932-00000081

[B95] Lupi C. Morin H. Deslauriers A. Rossi S. (2010). Xylem phenology and wood production: resolving the chicken-or-egg dilemma. Plant Cell Environ. 33, 1721–1730. 10.1111/j.1365-3040.2010.02176.x 20525004

[B96] Maceda A. Soto-Hernández M. Peña-Valdivia C. B. Terrazas T. (2018). Chemical composition of cacti wood and comparison with the wood of other taxonomic groups. Chem. Biodivers. 15, 4. 10.1002/cbdv.201700574 29444386

[B97] Malavasi B. C. Davis A. S. Malavasi M. M. (2016). Lignin in wood plants under water stress: a review. Floresta Ambient. 23, 589–597. 10.5061/dryad.ps837

[B98] Marques A. V. Rencoret J. Gutiérrez A. del Río J. C. Pereira H. (2016). Ferulates and lignin structural composition in cork. Holzforschung 70, 275–289. 10.1515/hf-2015-0014

[B99] Martone P. T. Estevez J. M. Lu F. Ruel K. Denny M. W. Somerville C. (2009). Discovery of lignin in seaweed reveals convergent evolution of cell-wall architecture. Curr. Biol. 19, 169–175. 10.1016/j.cub.2008.12.031 19167225

[B100] Mauseth J. D. Plemons B. J. (1995). Developmentally variable, polymorphic woods in cacti. Am. J. Bot. 82, 1199–1205. 10.2307/2446241

[B101] Mauseth J. D. Plemons B. J. (1998). Evolution of extreme xeromporhic characters in wood: a study of nine evolutionary lines in Cactaceae. Am. J. Bot. 85, 209–218. 10.2307/2446308

[B102] Mauseth J. D. Uozumi Y. Plemons B. J. Landrum J. V. (1995). Structural and systematic study of an unusual tracheid type in cacti. J. Plant Res. 108, 517–526. 10.1007/BF02344242

[B103] Mauseth J. D. (2004). Wide-band tracheids are present in almost all species of Cactaceae. J. Plant Res. 117, 69–76. 10.1007/s10265-003-0131-5 14639504

[B104] Mauseth J. D. (2006). Structure function relationships in highly modified shoots of Cactaceae. Ann. Bot. 98, 901–926. 0.1093/aob/mcl133 16820405 10.1093/aob/mcl133 PMC2803597

[B105] McCully M. Canny M. Baker A. Miller C. (2014). Some properties of the walls of metaxylem vessels of maize roots, including tests of the wettability of their lumenal wall surfaces. Ann. Bot. 113, 977–989. 10.1093/aob/mcu020 24709790 PMC3997638

[B106] Menden B. Kohlhoff M. Moerschbacher B. M. (2007). Wheat cells accumulate a syringyl-rich lignin during the hypersensitive resistance response. Phytochemistry 68, 513–520. 10.1016/j.phytochem.2006.11.011 17188312

[B107] Mohammadkhani A. Stoddard F. L. Marshall D. R. (1998). Survey of amylose content in *Secale cereale*, *Triticum monococcum*, *T. turgidum* and *T. tauschii* . J. Cereal Sci. 28, 273–280. 10.1016/S0733-5210(98)90007-8

[B108] Monroy M. A. Peña-Valdivia C. García J. R. Solano E. (2017). Chemical scarification and ozone in seed dormancy alleviation of wild and domesticated *Opuntia*, Cactaceae. Ozone Sci. Eng. 39, 104–114. 10.1080/01919512.2016.1261010

[B109] Nerd A. Neumann P. M. (2004). Phloem water transport maintains stem growth in a drought-stressed crop cactus (*Hylocereus undatus*). J. Amer. Soc. Hortic. Sci. 129, 486–490. 10.21273/JASHS.129.4.0486

[B110] Novaes E. Kirst M. Chiang V. Winter-Sederoff H. Sederoff R. (2010). Lignin and biomass: a negative correlation for wood formation and lignin content in trees. Plant Physiol. 154, 555–561. 10.1104/pp.110.161281 20921184 PMC2949025

[B111] Obst J. R. Ralph J. (1983). Characterization of hardwood lignin: investigation of syringyl/guaiacyl composition by ^13^C nuclear magnetic resonance spectroscopy. Hozlfoschung 37, 297–302. 10.1515/hfsg.1983.37.6.297

[B112] Pandey K. K. (2005). Study of the effect of photo-irradiation on the surface chemistry of wood. Polym. Degrad. Stabil. 90, 9–20. 10.1016/j.polymdegradstab.2005.02.009

[B113] Park E. H. Kahng J. H. Lee S. H. Shin K. H. (2001). An anti-inflammatory principle from cactus. Fitoterapia. 72, 299–290. 10.1016/S0367-326X(00)00287-2 11295308

[B114] Pawlicka A. Waliszewska B. (2011). Chemical composition of selected species of exotic wood derived from the region of Africa. Acta Sci. Pol. Technol. Aliment. 10, 37–41. 10.5552/drind.2018.1733

[B115] Pereira L. Flores-Borges D. N. A. Bittencourt P. R. L. Mayer J. L. S. Kiyota E. Araújo P. (2018). Infrared nanospectroscopy reveals the chemical nature of pit membranes in water-conducting cells of the plant xylem. Plant Physiol. 177, 1629–1638. 10.1104/pp.18.00138 29871981 PMC6084671

[B116] Plavcová L. Jansen S. (2015). “The role of xylem parenchyma in the storage and utilization of nonstructural carbohydrates”, in Functional and ecological xylem anatomy. Ed. Hacke U. (Switzerland: Springer International Publishing), 209–234.

[B117] Popper Z. A. Michel G. Hervé C. Domozych D. S. Willats W. G. T. Tuohy M. G. (2011). Evolution and diversity of plant cell walls: from algae to flowering plants. Annu. Rev. Plant Biol. 62, 567–590. 10.1146/annurev-arplant-042110-103809 21351878

[B118] Pratt R. B. Jacobsen A. L. Ewers F. W. Davis S. D. (2007). Relationships among xylem transport, biomechanics and storage in stems and roots of nine Rhamnaceae species of the California chaparral. New Phytol. 174, 787–798. 10.1111/j.1469-8137.2007.02061.x 17504462

[B119] Prislan P. Koch G. Čufar K. Gričar J. Schmitt U. (2009). Topochemical investigations of cell walls in developing xylem of beech (*Fagus sylvatica* L.). Holzforschung. 63, 482–490. 10.1515/HF.2009.079

[B120] Pritchard H. N. Hall J. A. (1976). The chemical composition of glochids from *Opuntia* . Can. J. Bot. 54, 173–176. 10.1139/b76-016

[B121] Růžička K. Ursache R. Hejátko J. Helariutta Y. (2015). Xylem development – from the cradle to the grave. New Phytol. 207, 519–535. 10.1111/nph.13383 25809158

[B122] Radotić K. Kalauzi A. Djikanović D. Jeremić M. Leblanc R. M. Cerović Z. G. (2006). Component analysis of the fluorescence spectra of a lignin model compound. J. Photoch. Photobio. B. 83, 1–10. 10.1016/j.jphotobiol.2005.12.001 16406801

[B123] Reddy K. O. Maheswari C. U. Shukla M. Muzenda E. (2014). Preparation, chemical composition, characterization, and properties of Napier grass paper sheets. Sep. Sci. Technol. 49, 1527–1534. 10.1080/01496395.2014.893358

[B124] Ren H. Dai X. Zhai H. Liu Z. Omori S. (2015). Comparison of bamboo native lignin and alkaline lignin modified by phased-separation method. Cellulose Chem. Technol. 49, 429–438.

[B125] Renault H. Werck-Reichhart D. Weng J. K. (2019). Harnessing lignin evolution for biotechnological application. Curr. Opin. Biotechnol. 56, 105–111. 10.1016/j.copbio.2018.10.011 30439673

[B126] Reti L. Castrillón J. A. (1951). Cactus alkaloids. *Trichocereus terscheckii* (Parmentier) Britton and Rose. J. Am. Chem. Soc. 73, 1767–1769. 10.1021/ja01148a097

[B127] Reyes-Rivera J. Terrazas T. (2017). Lignin analysis by HPLC and FTIR, in Xylem: methods and protocols, methods in molecular biology. Eds. De-Lucas J. P. Etchells M. (Durham: Human Press), 193–211. 10.1007/978-1-4939-6722-3_14 28050837

[B128] Reyes-Rivera J. Canché-Escamilla G. Soto-Hernández M. Terrazas T. (2015). Wood chemical composition in species of Cactaceae: the relationship between lignification and stem morphology. PloS One 10, 4. 10.1371/journal.pone.0123919 PMC4399841 25880223

[B129] Reyes-Rivera J. Soto-Hernández M. Canché-Escamilla G. Terrazas T. (2018). Structural characterization of lignin in four cacti wood: implications of lignification in the growth form and succulence. Front. Plant Sci. 9, 1518. 10.3389/fpls.2018.01518 30386367 PMC6199501

[B130] Rojas-Aréchiga M. Mandujano M. C. (2013). Aspectos sobre la germinación de *Myrtillocactus geometrizans*, *Stenocereus dumortieri* y *Echinocereus cinerascens* . Cact. Suc. Mex. 58, 118–126.

[B131] Sánchez-Soto B. Reyes-Olivas A. García-Moya E. Terrazas T. (2010). Germinación de tres cactáceas que habitan la región costera del noroeste de México. Interciencia 35, 299–305.

[B132] Safou-Tchiama R. Barhé T. A. Soulounganga P. Akagah A. G. De Jeso B. (2017). A comparative study of the syringyl, guaiacyl and hydroxyl groups units distribution in some African tropical hardwoods lignin by Py-GC/MS and spectroscopic techniques. JMES. 8, 2530–2540. 10.1007/s13196-018-0222-5

[B133] Sahin H. T. Arslan M. B. (2008). A study on physical and chemical properties of cellulose paper immersed in various solvent mixtures. Int. J. Mol. Sci. 9, 78–88. 10.1515/chem-2016-0043 19325721 PMC2635603

[B134] Saito K. Watanabe Y. Shirakawa M. Matsushita Y. Imai T. Koike T. (2012). Direct mapping of morphological distribution of syringyl and guaiacyl lignin in the xylem of maple by time-of-flight secondary ion mass spectrometry. Plant J. 69, 542–552. 10.1111/j.1365-313X.2011.04811.x 21978273

[B135] Salas-Cruz L. R. Pournavab R. F. Díaz-Jiménez L. Hernández-Piñero J. L. Carrillo-Parra A. Cárdenas-Ávila M. L. (2014). Seed germination and seedling survival of six cacti species using natural zeolite as substrate. IJCRAR 2, 81–91. 10.3390/d10040121

[B136] Scheller H. V. Ulvskov P. (2010). Hemicelluloses. Annu. Rev. Plant Biol. 61, 263–289. 0.1146/annurev-arplant-042809-112315 20192742 10.1146/annurev-arplant-042809-112315

[B137] Shebani A. N. Reene A. J. Meincken M. (2009). The effect of wood extractives on the thermal stability of different Wood-LLDPE composites. Thermochim. Acta 481, 52–56. 10.1016/j.tca.2008.10.008

[B138] Skyba O. Douglas C. J. Mandsfield S. D. (2013). Syringyl-rich lignin renders poplars more resistant to degradation by wood decay fungi. Appl. Environ. Microbiol. 79, 2560–2571. 10.1128/AEM.03182-12 23396333 PMC3623167

[B139] Souza L. F. Gasparetto B. F. Lopes R. R. Barros I. B. I. (2016). Temperature requirements for seed germination of *Pereskia aculeata* and *Pereskia grandiflora* . J. Therm. Biol. 57, 6–10. 10.1016/j.jtherbio.2016.01.009 27033034

[B140] Speranza M. Gutiérrez A. del Río. J. C. Bettucci L. Martínez A. T. Martínez M. J. (2009). Sterols and lignin in *Eucalyptus globulus* Labill. Wood: spatial distribution and fungal removal as revealed by microscopy and chemical analyses. Holzforschung 63, 362–370. 10.1515/HF.2009.041

[B141] Sperry J. S. Hacke U. G. Field T. S. Sano Y. Sikkema E. H. (2007). Hydraulic consequences of vessel evolution in Angiosperms. Int. J. Plant Sci. 168, 1127–1139. 10.1086/520726

[B142] Takabe K. Miyauchi S. Tsunoda R. Fukazawa K. (1992). Distribution of guaiacyl and syringyl lignins in japanese beech (*Fagus crenata*): variation within an anual ring. IAWA J. 13, 105–112. 10.1163/22941932-90000561

[B143] Tutt M. Olt J. (2011). Suitability of various plant species for bioethanol production. Agron. Res. Biosystem Eng. Special Issue 1, 261–267.

[B144] Vázquez-Sánchez M. Terrazas T. (2011). Stem and wood allometric relationships in Cacteae (Cactaceae). Trees. 25, 755–767. 10.1007/s00468-011-0553-y

[B145] Vázquez-Sánchez M. Terrazas T. Grego-Valencia D. Arias S. (2017). A growth form and wood evolution in the tribe Cacteae (Cactaceae). Willdenowia 47, 49–67. 10.3372/wi.47.47106

[B146] Vargas-Muñoz J. O. (2008).Comportamiento de algunos extractos de la corteza de pino Caribe (Pinus caribaea Morelet var. Hondurensis Barret & Golfari) sobre crecimiento de hongos xilófagos y su acción antioxidante. Cochabamba: Centro de Interacción Social e Información Forestal-CISIFOR.

[B147] Vena P. F. Görgens J. F. Rypstra T. (2010). Hemicelluloses extractions from giant bamboo prior to kraft and soda AQ pulping to produce paper pulps, value-added biopolymers and bioethanol. Cell Chem. Technol. 44, 153–163. 10.1515/hf-2012-0197

[B148] Wahab R. Mustafa M. T. Sudin M. Mohamed A. Rahman S. Samsi H. W. (2013). Extractives, hollocellulose, α-cellulose, lignin and ash contents in cultivated tropical bamboo *Gigantochloa brang*, *G. levis*, *G. scortechinii* and *G. wrayi* . Curr. Res. J. Biol. Sci. 5, 266–272. 10.5539/jas.v5n8p66

[B149] Watanabe Y. Kojima Y. Ona T. Asada T. Sano Y. Fukazawa K. (2004). Histochemical study on heterogeneity of lignin in *Eucalyptus* species II. The distribution of lignins and polyphenols in the walls of various cell types. IAWA J. 25, 283–295. 10.1163/22941932-90000366

[B150] Weng J. K. Chapple C. (2010). The origin and evolution of lignin biosynthesis. New Phytol. 187, 273–285. 10.1111/j.1469-8137.2010.03327.x 20642725

[B151] Weng J. K. Akiyama T. Bonawitz N. D. Li X. Ralph J. Chapple C. (2010). Convergent evolution of syringyl lignin biosynthesis via distinct pathways in the Lycophyte *Selaginella* and flowering plants. Plant Cell. 22, 1033–1045. 10.1105/tpc.109.073528 20371642 PMC2879749

[B152] Yoshizawa N. Watanabe N. Yokota S. Idei T. (1993a). Distribution of guaiacyl and syringyl in normal and compression wood of *Buxus microphylla* var. *insularis* Nakai. IAWA J. 14, 139–151. 10.1163/22941932-90001307

[B153] Yoshizawa N. Satoh M. Yokota S. Idei T. (1993b). Formation and structure of reaction wood in *Buxus microphylla* var. *insularis* Nakai. Wood. Sci. Technol. 27, 1–10. 10.1007/BF00203405

[B154] Yoshizawa N. Ohba H. Uchiyama J. Yokota S. (1999). Deposition of lignin in differentiating xylem cell walls of normal and compression wood of *Buxus microphylla* var. *insularis* Nakai. Holzforshung. 53, 156–160. 10.1515/HF.1999.026

[B155] Zeng Y. Zhao S. Wei H. Tucker M. P. Himmel M. E. Mosier N. (2015). In situ micro-spectroscopic investigation of lignin in poplar cell walls pretreated by maleic acid. Biotechnol. Biofuels 8, 126. 10.1186/s13068-015-0312-1 26312066 PMC4549890

[B156] Zeng Y. Himmel M. E. Ding S.-Y. (2017). Visualizing chemical functionality in plant cell walls. Biotechnol. Biofuels 10, 263. 10.1186/s13068-017-0953-3 29213316 PMC5708085

[B157] Zhang J. Choi Y. S. Yoo C. G. (2015). Cellulose-hemicellulose and cellulose-lignin interactions during fast pyrolysis. ACS Sustain. Chem. Eng. 3, 293–301. 10.1021/sc500664h

[B158] Zhou C. Li Q. Chiang V. L. Lucia L. A. Griffs D. P. (2011a). Chemical and spatial differentiation of syringyl and guaiacyl lignins in Poplar wood via Time-of-flight secondary Ion Mass Spectrometry. Anal. Chem. 83, 7020–7026. dx..org/10.1021/ac200903y 21851065 10.1021/ac200903y

[B159] Zhou G. Taylor G. Polle A. (2011b). FTIR-ATR-based prediction and modelling of lignin and energy contents reveals independent intra-specific variation of these traits in bioenergy poplars. Plant Methods 7, 9. 10.1186/1746-4811-7-9 21477346 PMC3094334

