# Supplementary figures and images for: Differences in the Structural Chemical Composition of the Primary Xylem of Cactaceae: A Topochemical Perspective
Source: Front Plant Sci. 2019 Nov 28;10:1497. doi: 10.3389/fpls.2019.01497 (PMC6892835; doi:10.3389/fpls.2019.01497)

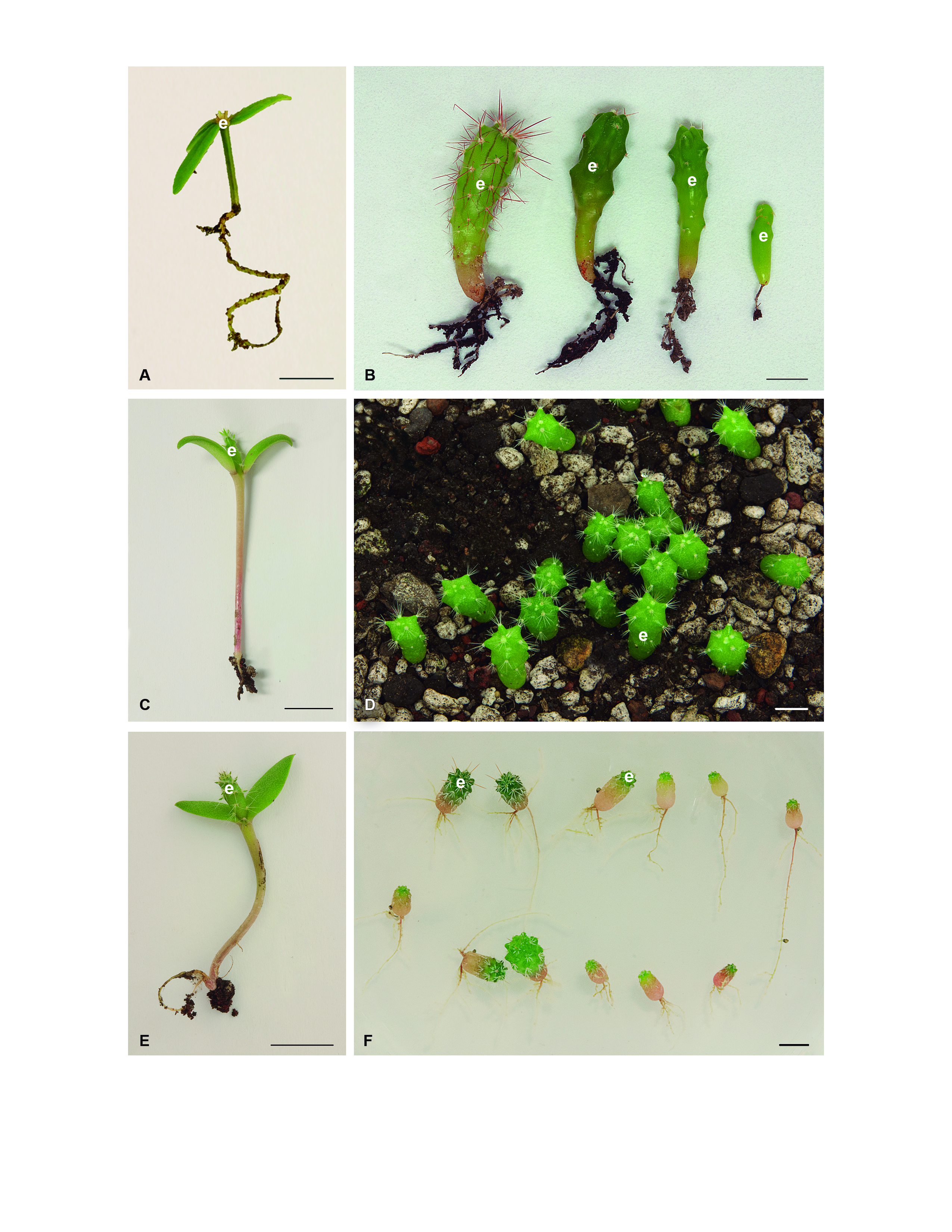

Supplement: Supplementary file 1 [file Image_1.tif]
