# Supplementary material for: Differences in the Structural Chemical Composition of the Primary Xylem of Cactaceae: A Topochemical Perspective
Source: Front Plant Sci. 2019 Nov 28;10:1497. doi: 10.3389/fpls.2019.01497 (PMC6892835; doi:10.3389/fpls.2019.01497)
Supplement: Supplementary file 2 [file Table_1.docx]

Supplementary Material

Table S1. Percentages of the extractives and lignocellulosic compounds of species with primary xylem.

| **Species** | **Extractives**  **%** | **Cellulose**  **%** | **Hemicellulose**  **%** | **Lignin**  **%** | **References** |
| --- | --- | --- | --- | --- | --- |
| *Bambusa vulgaris* | 6.79 | 47.51 | 24.44 | 21.26 | Ekebafe et al., 2011 |
| *Bromus inermis* | - | 30 | 28.2 | 34.7 | Bidlack and Buxton, 1992 |
| *Cannabis sativa* | - | 53.86 | 10.6 | 8.76 | Tutt and Olt, 2011 |
| *Dactylis glomerata* | 39.2-56.9 | 23.6-52.3 | 23.5 | 21.3 | Bidlack and Buxton, 1992 |
| *Dendrocalamus giganteus* | 7.1 | 44.4 | 25-35 | 22 | Vena et al., 2010 |
| *D. oldhami* | 3.3-3.9 | 34.1-47.1 | 25.1-34.5 | 20.9-25.2 | Cao et al., 2014 |
| *Eleusine indica* | - | 26.1 | 32.51 | 4.14 | Ekpo et al., 2016 |
| *Festuca arundinacea* | 33.7-36.2 | 26.7-28.4 | 30.3-31.9 | 43-49 | Cherney et al., 1988 |
| *Gigantochloa brang* | 8-9.23 | 34-52 | - | 25-33 | Wahab et al., 2013 |
| *Helianthus annuus* | - | 34.06 | 5.18 | 7.72 | Tutt and Olt, 2011 |
| *H. tuberosus* | - | 20.95 | 5.48 | 5.05 | Tutt and Olt, 2011 |
| *Lotus corniculatus* | 49.7-54.5 | 22.7-28.7 | 13.4-25.5 | 9.2-30 | Cherney et al., 1988; Bidlack and Buxton, 1992. |
| *Medicago sativa* | 48.9-55.2 | 14.7-23.9 | 13-15.1 | 8.2-17.4 | Cherney et al., 1988; Bidlack and Buxton, 1992. |
| *Miscanthus saccharifloris* | - | 42 | 30.15 | 9.65 | Tutt and Olt, 2011 |
| *Neosinocalamus affinis* | 4.5 | - | 62.4 | 24.3 | Ren et al., 2015 |
| *Panicum maximum* | - | 32.71 | 42.57 | 3.09 | Ekpo et al., 2016 |
| *P. virgatum* | 24.9-27.1 | 32.2-43 | 33.6-36.4 | 46.1-68 | Cherney et al., 1988; Bidlack and Buxton, 1992. |
| *Pennisetum purpureum* | - | 34.04-46.58 | 34.14-39.31 | 5.62-22.25 | Kamarullah et al., 2015; Ekpo et al., 2016 |
| *Phalaris arundinacea* | 33.3-38.1 | 25.5-27 | 30.3-33.1 | 45-48 | Cherney et al., 1988 |
| *Phyllostachys edulis* | - | 44.63 | 23.65 | 20.35 | Li et al., 2015 |
| *Secale cereale* | 26.4-28.6 | 34.4-34.8 | 28.2-30.2 | 6.4-6.9 | Cherney et al., 1988 |
| *Sorghum bicolor* | 38.2-43.4 | 25.6-28.5 | 26.4-27.7 | 4.6-5.3 | Cherney et al., 1988 |
| *Trifolium pratense* | - | 25 | 20.6 | 34 | Bidlack and Buxton, 1992 |
| *Zea mays* | 6.6 | 39.27 | 25.96-64.2 | 9.02-19.9 | Tutt and Olt, 2011; Zhang et al., 2015 |

Table S2. Kruskal-Wallis values for each variable.

| **Variables** | **Chi-cuadrado** | **Degrees of freedom** | **Probability>Chi-square** |
| --- | --- | --- | --- |
| Ethanol-benzene | 25.69 | 7 | 0.001*** |
| Ethanol | 24.36 | 7 | 0.001*** |
| Water 90 °C | 25.29 | 7 | 0.001*** |
| Total extractives | 25.84 | 7 | 0.001*** |
| Extractives-free wood | 25.84 | 7 | 0.001*** |
| Celullose | 15.95 | 7 | 0.026* |
| Lignin | 22.36 | 7 | 0.002*** |
| Hemicellulose | 12.04 | 7 | 0.099 |

***= highly significant differences; *=significant differences.

Figure S1. Seedlings of Cactaceae. A. *Leuenbergeria lychnidiflora*, eight months. B. *Echinocactus platyacanthus*, 1-8 months. C. *Cylindropuntia imbricata*, eight months. D. *Ferocactus pilosus*, eight months. E. *Opuntia streptacantha*, eight months. F. *Mammillaria carnea*, 2-8 months. Bar is 1 cm in A-E; 5 mm in F. e=epicotyl (=stem).
